# Transcriptome Analysis Did Not Show Endogenous Stem Cell Characteristics in Murine Lgr5^+^ Retinal Cells

**DOI:** 10.3390/ijms20143547

**Published:** 2019-07-19

**Authors:** Carolyn Trepp, Ana Maria Quintela Pousa, Volker Enzmann

**Affiliations:** 1 F. Hoffmann-La Roche, 4070 Basel, Switzerland; 2 Department of Ophthalmology, University Hospital of Bern, University of Bern, 3010 Bern, Switzerland; 3 Department for BioMedical Research, University of Bern, 3008 Bern, Switzerland

**Keywords:** Lgr5^+^ cells, stem cells, amacrine cells, interneurons, retinal development, transcriptomics

## Abstract

Lgr5, an intestinal adult stem cell marker, was recently also found in neuronal tissues. We investigated whether retinal Lgr5^+^ cells express properties of neural stem cells (NSC) and/or of differentiated interneurons during retinal development. RNA was isolated from Lgr5^+^ and Lgr5^−^ populations from postnatal day 5 (PN5) and adult retinas of Lgr5^EGFP-Ires-CreERT2^ knock-in mice sorted by fluorescence-activated cell sorting (FACS). Transcriptome analyses were performed on two RNA samples of each developmental stage (PN5 and adult). The online platform PANTHER (Protein ANalysis THrough Evolutionary Relationships) was used to determine overrepresented gene ontology (GO) terms of biological processes within the set of differentially expressed genes. The detailed evaluation included gene expression in regard to stem cell maintenance/proliferation, cell cycle, and Wnt signaling but also markers of differentiated retinal neurons. None of the enriched GO terms of upregulated genes of Lgr5^+^ cells showed a positive association to NSC. On the contrary, NSC maintenance and proliferation rather prevail in the Lgr5^−^ cell population. Furthermore, results suggesting that Wnt signaling is not active in the Lgr5^+^ population. Therefore, our transcriptome analysis of Lgr5^+^ retinal cells suggest that these cells are differentiated neurons, specifically glycinergic amacrine cells.

## 1. Introduction

Lgr5 (leucine-rich repeat containing G-protein receptor 5) belongs to a family of G-protein-coupled receptors [1] and is a Wnt target gene, which specifically acts as a receptor for Wnt agonists called R-spondins. Wnt signaling is critical during development, controlling cell fate decisions and tissue patterning [2]. In the adult organism, Wnt signals regulate homeostatic regeneration in numerous tissues, including the retina [3]. Furthermore, aberrant Wnt signaling is implicated in several diseases, especially cancer. It is classified into two pathways, GSK-3β dependent (canonical) and GSK-3β independent (non-canonical) [4]. In both cases, Wnt binds to the membrane receptor Frizzled (Fz). In the canonical pathway, Wnt binds to Fz and its co-receptor low-density lipoprotein receptor-related protein 5/6 (LRP5/6). Disheveled (Dvl) is recruited to the membrane resulting in the endocytosis of the complex to form a signalosome. The β-catenin destruction complex consisting of Axin2, adenomatous polyposis coli (APC), casein kinase-1α (CK-1α), and glycogen synthase kinase-3 (GSK-3β) is sequestered and disassembled. The inactivation of the destruction complex hinders GSK-3β in phosphorylating β-catenin. Thus, β-catenin evades proteasomal degradation and accumulates in the cytosol. Subsequently, β-catenin translocates to the nucleus, displaces the transcriptional repressor Groucho and bind to the T-cell factor/lymphoid-enhancing factor (TCF/LEF) promoter/enhancer complex. Thereby, the transcription of Wnt target genes, such as Lgr5, cyclinD1, c-Myc and Axin2, is activated. Lgr5 binding to the receptor for R-spondin enhances LRP6 phosphorylation through the canonical Wnt pathway [5].

Lgr5 was first identified as an adult stem cell marker in the intestine [6]. Lineage-tracing experiments demonstrated that Lgr5^+^ cells give rise to all types of intestinal cells. Subsequently, Lgr5 was found to be expressed in several other adult stem cell populations such as the stomach, the hair follicle, and taste buds [7]. Furthermore, alternative splicing is common in the Lgr-family with three splice variants known for Lgr5: Lgr5∆5 lacking exon 5, Lgr5∆8 lacking exon 8, and Lgr5∆5–8 lacking exons 5–8 [8]. All Lgr5 transcript variants might be involved in the EMT process through activating the Wnt-signaling pathway [9]. It has been shown that Lgr5^+^ cells can be expanded in vitro and can differentiate into different cell types of the tissue of origin [10,11]. In the murine retina, Lgr5 is expressed in glycinergic amacrine interneurons [12]. Even though Lgr5^+^ cells demonstrate properties of differentiated interneurons, they have been shown to contribute to the generation of new retinal cells in adult animals [1]. In this study, we investigated whether Lgr5^+^ cells harbor stem cell potential through the transcriptome analysis of postnatal day 5 (PN5) and adult retinas of Lgr5^EGFP-Ires-CreERT2^ knock-in mice.

## 2. Results

### 2.1. Lgr5^+^ Transcriptomics

#### RNA Quantity and Quality

Retinae of at least three GFP^+^ animals were pooled for FACS. GFP^+^ cells and 1 × 10^6^ cells of the negative fraction were collected for subsequent RNA isolation. Number of sorted cells, concentration of isolated RNA, total RNA, and the measured RNA quality index (RQI) of each sample is listed in Supplementary Table S1. Higher numbers of Lgr5^+^ cells were isolated from adult retinae compared to PN5. RNA yield varied markedly between isolations. Sample 5 showed the lowest yield of 4.27 ng RNA. The RNA concentration of this sample was too low to determine the RQI. However, electropherogram and virtual gel show two distinct peaks/bands of ribosomal RNA (18S and 28S) indicating good RNA quality (Supplementary Figure S1). All other samples revealed high RQI of above 9.

### 2.2. Quality Control of Microarray Processing

Transcriptome expression profiling was performed using Affymetrix Clariom S Mouse array chips. All samples demonstrated increasing signal values of hybridization controls (bioB < bioC < bioD < cre; Supplementary Figure S2A). In addition, consistent area under the ROC curve (AUC) values of between 0.8 and 0.85 were detected across all samples (Supplementary Figure S2B). Hence, all samples passed quality assessment. Moreover, signal box plots showed similar distributions of log2-transformed signal values (Supplementary Figure S2C).

Principle component analysis (PCA) is used to reduce the complexity of high-dimensional data while retaining information that is responsible for the variance between datasets. Samples with similar transcriptional profiles will cluster whereas samples with the highest variance will show the highest distance between each other.

PCA of the eight analyzed samples showed that biological replicates clustered according to their developmental stage and Lgr5-expression (Supplementary Figure S3). Furthermore, the highest variance in transcriptional profiles was found between Lgr5^+^ and Lgr5^−^ cells of different developmental stages (adult Lgr5^+^ and PN5 Lgr5^−^; adult Lgr5^−^ and PN5 Lgr5^+^).

### 2.3. Differential Gene Expression (DGE) and PANTHER Analysis

DGE of Lgr5^+^ cells of the two developmental stages was investigated. For both adult and PN5, gene expression values of Lgr5^+^ cells were compared to Lgr5^−^ cells. Furthermore, DGE of PN5 compared to adult Lgr5^+^ cells were analyzed. Differentially expressed genes were defined by a fold change ≥ 2 or ≤ −2, *p*-value < 0.05, and false discovery rate (FDR) < 0.05. The online tool PANTHER was used to determine overrepresented gene ontology (GO) terms of biological processes within the set of differentially expressed genes. Thereby, the input list was compared to the list of all genes analyzed by the microarray and the fold enrichment was calculated.

**Figure 1:**
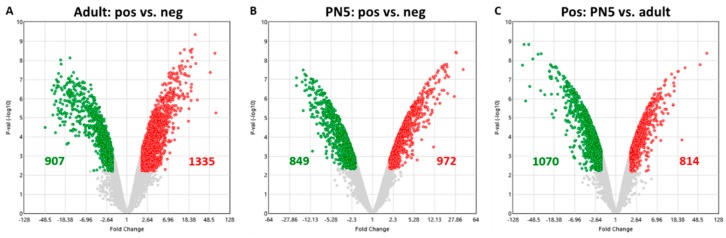
Volcano plots of the up- and downregulated DEG in (**A**) adult Lgr5^+^ compared to adult Lgr5^−^, (**B**) PN5 Lgr5^+^ compared to PN5 Lgr5^−^ and (**C**) PN5 Lgr5^+^ compared to adult Lgr5^+^. The horizontal axis is fold change ≥ 2 or ≤ −2, and the vertical axis represents the *p*-value after the negative logarithm conversion. The red dots indicate the upregulated genes, whereas the green dots represent the downregulated genes. The corrected *p* < 0.05 was an absolute threshold used to select DEGs. DEGs, differentially expressed genes.

The Clariom S Mouse array analyzes a total of 22,206 genes. Of these, 2242 genes were differentially expressed in adult Lgr5^+^ compared to adult Lgr5^−^, 1335 being upregulated and 907 being downregulated (Figure 1A). At PN5, Lgr5^+^ presented 1821 differentially-expressed genes compared to Lgr5^−^. Thereof, 972 genes showed significantly higher whereas 849 showed significantly lower expression compared to the negative populations (Figure 1B). When comparing the positive populations of both developmental stages, significant differences in gene expression was found in 1884 genes. Thereof, 814 genes were upregulated and 1070 downregulated (Figure 1C).

The lists of up- or downregulated genes from DGE were used as input lists for the statistical enrichment analysis by PANTHER. Due to the high number of overrepresented GO terms, only the top ten overrepresented terms with the highest fold enrichments (FE) of upregulated (+) and downregulated (−) genes are shown (Table 1, Table 2 and Table 3). Complete lists of all analyses can be found in the supplementary materials section (Supplementary Tables S2–S7).

From upregulated genes in adult Lgr5^+^, GO terms related to synaptic function were the most prevalent. Around a third of all enriched GO terms including seven of the top ten were associated to either pre- or postsynaptic organization, neurotransmitter signaling, action potential or synaptic plasticity. The GO term “Synaptic vesicle clustering” showed the highest FE (16.00; *p* = 6.89 × 10^−4^; Table 1). Glycinergic, GABAergic, glutamatergic, as well as dopaminergic signaling showed significant overrepresentation. Yet, glycinergic signaling showed the highest FE of all neurotransmitters (“Synaptic transmission, glycinergic”; FE = 11.43; *p* = 3.91 × 10^−4^). Furthermore, genes related to neuronal morphology, like dendrite and axon morphogenesis, were enriched. Downregulated genes in adult Lgr5^+^ showed an enrichment for GO terms associated to phototransduction, photoreceptor cell differentiation, and maintenance.

Approximately half of the overrepresented GO terms of upregulated genes in PN5 Lgr5^+^ cells were associated to synaptic signaling. Negative regulation of dopamine secretion showed the highest FE (Table 2; FE = 23.51, *p* = 2.37 × 10^−5^). The highest FE of a positive reference to neurotransmitter signaling was the GO term “Positive regulation of AMPA receptor activity” (FE = 13.06; *p* = 1.64 × 10^−4^) indicating glutamatergic inputs to Lgr5^+^ cells. Yet, the GO term “Glycine transport” (FE = 11.76, *p* = 1.2 × 10^−2^) showed the highest FE for a presynaptic neurotransmitter. In addition, genes for GABAergic and cholinergic synaptic transmission showed significant enrichment. Like in adult Lgr5^+^, genes related to neuronal morphology were also enriched. Of the 158 overrepresented GO terms of downregulated genes, “Retinal rod cell differentiation” showed the highest FE (Supplementary Table S5; FE = 24.18; *p* = 1.52 × 10^−4^). Several terms associated to cell proliferation, such as cell cycle progression, DNA replication, chromatin segregation and cytokinesis, were found (Supplementary Table S5). Additionally, genes associated to the terms “neuronal stem cell maintenance” and “positive regulation of neural precursor cell proliferation” were significantly enriched with FE of 6.31 (*p* = 8.35 × 10^−4^) and 3.45 (*p* = 4.2 × 10^−4^), respectively (Supplementary Table S5). Furthermore, genes related to angiogenesis and the regulation of apoptosis were enriched.

Comparing Lgr5^+^ cells from PN5 to adult animals, three major groups of overrepresented terms of upregulated genes were found. One consisting of terms related to cell cycle processes, such as the term with the second highest FE “DNA damage response, signal transduction by p53 class mediator resulting in transcription of p21 class mediator” (Table 3; FE = 12.11; *p* = 1.92 × 10^−4^) and different cell cycle phase transitions. The second group encompasses terms associated to development and neuronal differentiation. For example, “Regulation of development, heterochronic” (FE = 8.88; *p* = 6.04 × 10^−4^). The last group includes terms connected to transcriptional and translational processes, with “ribosomal small subunit assembly”, “cytoplasmic translation”, and “ribosomal large subunit assembly” all in the top ten of overrepresented terms. Furthermore, genes associated to the term “positive regulation of Notch signaling pathway” showed significant enrichment (FE = 5.33; *p* = 2.97 × 10^−4^). The 77 overrepresented GO terms of downregulated genes in Lgr5^+^ cells at PN5 were mostly related to synaptic signaling and transmission of nerve impulse. Synaptic signaling includes GO terms of synapse assembly, plasticity, as well as synaptic transmission. Whereas transmission of nerve impulse encompasses processes of action potential generation, hence the transport of different ions across the plasma membrane, regulation of intracellular ion concentrations and membrane repolarization.

### 2.4. Gene List Analysis

For better understanding of the PANTHER overrepresentation analysis, specific gene lists were analyzed for expression values and fold changes of Lgr5^+^ cells. The list of up- or downregulated genes of DGE is the input for the PANTHER tool. It does not take signal values into account. Therefore, genes that display low signal values of below 100 but show significant differential gene expression compared to the control population will still be analyzed by PANTHER.

Lgr5 marks stem cell populations in several tissues. Furthermore, it has been proposed that retinal Lgr5^+^ cells harbor regenerative potential. Regarding genes involved in NSC maintenance, in the PANTHER analysis only Prox1 was highly expressed in both PN5 and adult, showing mean values of 1138 and 889, respectively (Supplementary Figure S4).

**Figure 2:**
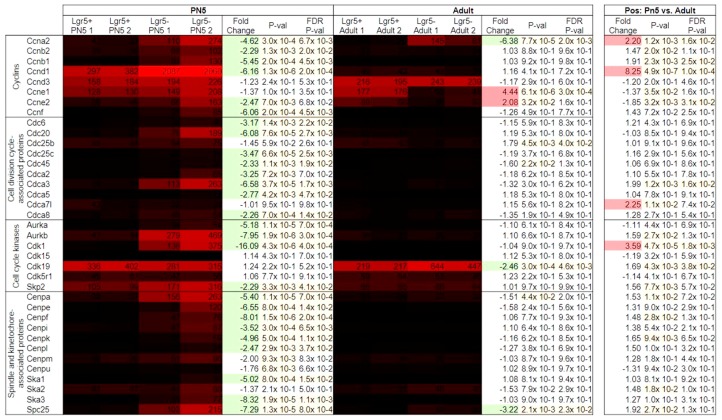
Gene expression levels and fold changes of cell cycle markers. Fold change describes downregulated cell cycle genes at PN5 in Lgr5^+^ cells compared to Lgr5^−^ cells. Differentially expressed genes were defined by a fold change ≥ 2 or ≤ −2, *p*-value < 0.05, and false discovery rate (FDR) < 0.05.

On the other hand, our analysis revealed that most of the analyzed cell cycle genes were downregulated in PN5 Lgr5^+^ cells (Figure 2). However, signal values of these genes were relatively low, mostly < 100. Still, confirming the PANTHER analysis, some of these genes showed significantly higher values than expression in adult Lgr5^+^ cells. For example, Cdk1 showed the highest fold change in signal values between PN5 Lgr5^+^ and PN5 Lgr5^−^ (FC: −16.09; *p* = 4.3 × 10^−6^) with a mean signal intensity of 14 in positive cells. Furthermore, this was detected to be significantly higher than the signal value of Cdk1 in adult Lgr5^+^ cells (FC: 3.59; *p* = 4.7 × 10^−5^) with mean signal of 4. Of the analyzed cell cycle genes, the highest expressed gene was Ccnd1 in PN5 Lgr5^−^ cells.

**Figure 3:**
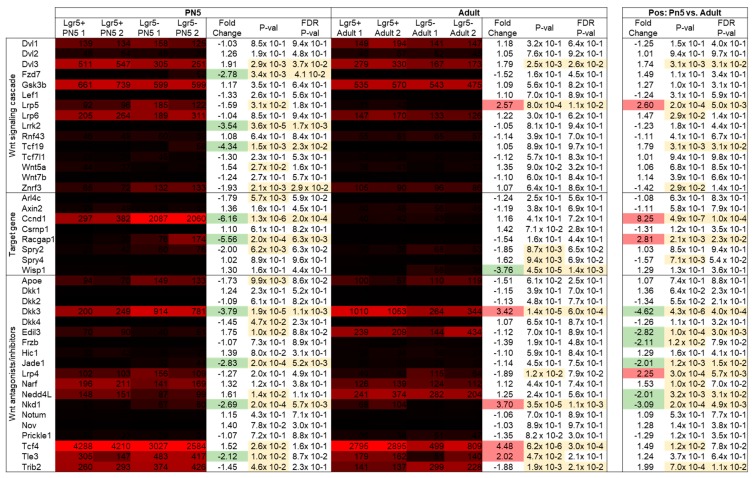
Gene expression levels and fold changes of Wnt-pathway members. Fold change shows lower expression of Wnt signaling genes at PN5 in Lgr5^+^ cells compared to Lgr5^−^ cells. Differentially expressed genes were defined by a fold change ≥ 2 or ≤ −2, *p*-value < 0.05, and false discovery rate (FDR) < 0.05.

Lgr5 is part of the Wnt signaling cascade. Yet, the only overrepresented GO term related to Wnt signaling in the PANTHER analysis was “negative regulation of canonical Wnt signaling pathway” (FE: 3.51, *p* = 2.3 × 10^−5^; Supplementary Table S5). The gene expression levels of members of the signaling cascade, target genes and Wnt antagonists were also investigated. At PN5 differentially expressed genes of the signaling cascade showed low signal intensities with mean values of below 40 (Figure 3). The Wnt target gene Ccnd1 is a marker of cell cycle progression. As mentioned above Ccnd1 showed significantly lower expression in Lgr5^+^ compared to Lgr5^−^ cells at PN5 (FC: −6.16, *p* = 1.6 × 10^−6^). Yet, Lgr5^+^ cells showed lower expression of Wnt signaling antagonists, for example Dkk3 (FC: −3.79, *p* = 1.9 × 10^−5^). In contrast, these genes were significantly higher expressed in adult Lgr5^+^ compared to Lgr5^−^ cells (Dkk1; FC: 3.42; *p* = 1.4 × 10^−5^).

**Figure 4:**
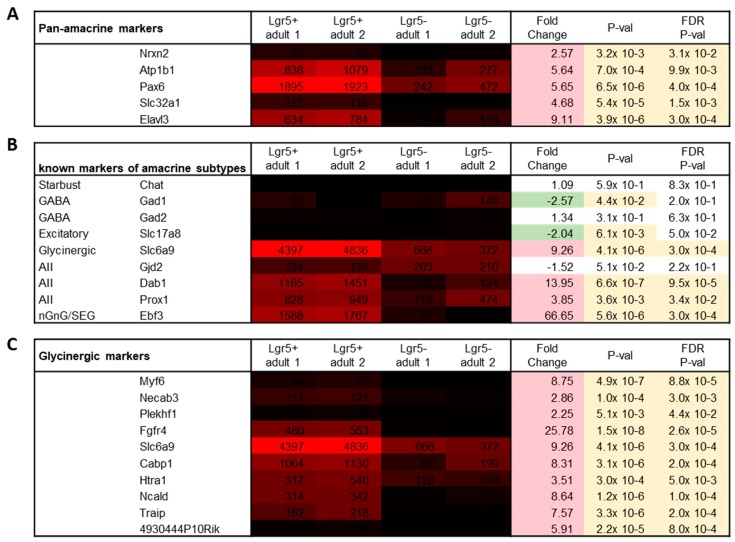
Gene expression levels and fold changes of amacrine cell markers in adult tissue. (**A**) Fold change shows higher expression of all pan-amacrine markers in adult Lgr5^+^ compared to Lgr5^−^ cells, (**B**) Slc6a9 marking glycinergic cells is the highest expressed of all known makers of amacrine subtypes, and (**C**) besides Slc6a9, other markers of glycinergic amacrine cells are also expressed in Lgr5^+^ cells. Differentially expressed genes were defined by a fold change ≥ 2 or ≤ −2, *p*-value < 0.0, and false discovery rate (FDR) < 0.05.

Adult Lgr5^+^ cells have been characterized as glycinergic amacrine cells. By morphological analysis they have been categorized as narrow-field amacrine cells, in particular as AII amacrine cells [10]. Yet, molecular profiling of Lgr5^+^ is lacking. Therefore, gene expression levels of different known amacrine markers were analyzed. With more than 40 different subtypes, amacrine cells represent one of the most diverse neuronal classes. Still several markers have been identified to be expressed across amacrine subtypes. Lgr5^+^ cells showed significantly higher levels of all analyzed pan-amacrine markers compared to the negative population (Figure 4A). The difference in expression levels varied greatly, the least expressed being Nrxn2 (mean: 88.3) and the highest Pax6 (mean: 1909). In addition, Slc32a1 showed low signal values (mean: 117).

Furthermore, molecular markers of the more widely studied amacrine cells have emerged. Lgr5^+^ cells showed signal values of <100 for all markers of GABAergic amacrine cells (Figure 4B; Chat, Gad1, and Gad2). Likewise, Lgr5^+^ did not express the marker of excitatory amacrine cells Slc17a (mean: 4). Except for Gjd2, genes of glycinergic subtypes (Slc6a9, Dab1, Prox1) were highly expressed. Moreover, Ebf3 showed high expression values in Lgr5^+^ cells. Ebf3 marks non-GABAergic non-glycinergic (nGnG) amacrine cells as well as Satb2-Ebf3-Glyt1-postive (SEG) amacrine cells. All markers of glycinergic amacrine cells were significantly higher expressed in Lgr5^+^ compared to Lgr5^−^ (Figure 4C). However, Myf6, Plekhf1 and 4930444P10Rik showed signal values < 100.

Since adult Lgr5^+^ cells highly expressed the nGnG and SEG amacrine cell marker Ebf3, other markers of nGnG cells were analyzed. The expression values of these markers were investigated in PN5 Lgr5^+^ cells since the transcriptional profile of nGnG cells has so far only been studied at PN6 [13].

**Figure 5:**
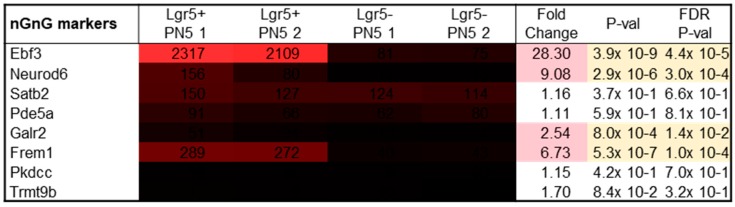
Gene expression levels and fold changes of nGnG cell markers in PN5 tissue. Fold change shows high expression values of Ebf3 in PN5 Lgr5^+^ cells. Differentially expressed genes were defined by a fold change ≥ 2 or ≤ −2, *p*-value < 0.05, and false discovery rate (FDR) < 0.05.

In addition, at PN5 Lgr5^+^ cells highly expressed Ebf3 (Figure 5; mean: 2213). Neurod6 showed a significant fold change of 9.08 in Lgr5^+^ compared to Lgr5^−^ (*p* = 2.9 × 10^−6^). Yet, the signal values are low compared to the expression of Ebf3 (mean: 118). Similar expression values were seen for Satb2 (mean: 138), however, these were not significantly upregulated compared to Lgr5^−^. In addition to Ebf3, Frem1 showed the highest signal values (mean: 280) and a significant fold change of 6.73 (*p* = 5.3 × 10^−7^).

**Figure 6:**
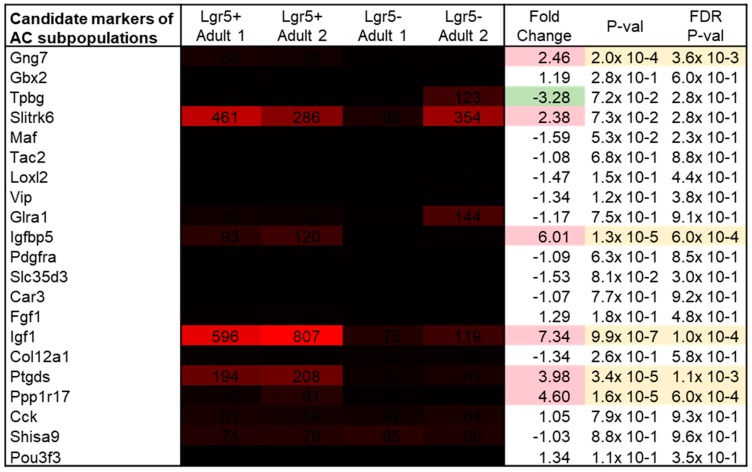
Gene expression levels and fold changes of candidate markers of amacrine cell subpopulations. Fold change shows expression of Slitrk6, Igf1 and Ptgds above the threshold of 100. Differentially expressed genes were defined by a fold change ≥ 2 or ≤ −2, *p*-value < 0.05, and false discovery rate (FDR) < 0.05.

By single cell RNA-sequencing Macosko et al. discovered candidate markers of amacrine subpopulations [14]. Therefore, expression of these markers in Lgr5^+^ cells was analyzed. The highest expression and significant fold change was seen for the marker Igf1, with a mean signal value of 701 and fold change of 7.34 (Figure 6; *p* = 9.9 × 10^−7^). Two other markers showed signal values of above 100 and significant upregulation, namely Ptgds (mean: 201, FC: 6.73, *p* = 5.3 × 10^−7^) and Igfbp5 (mean: 106; FC: 6.01, *p* = 1.3 × 10^−5^).

Amacrine cells are classified by the neurotransmitter they secret. Yet to integrate and modulate the visual signal they receive signals from different bipolar cells and other amacrine cells. For instance, the AII amacrine cell has been shown to receive at least nine different inputs arising from various cells and using diverse neurotransmitters [15]. Therefore, Lgr5^+^ cells were further characterized by their expression pattern of different neurotransmitter receptors.

**Figure 7:**
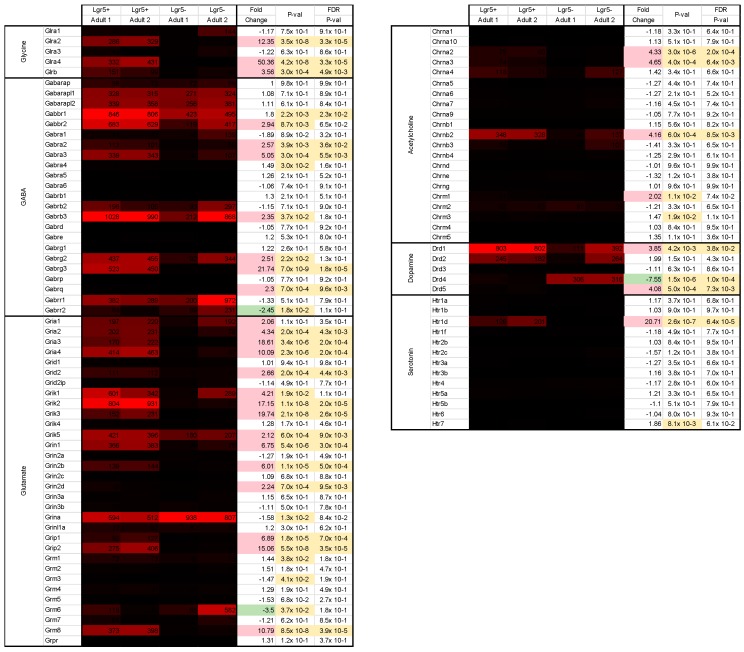
Gene expression levels and fold changes of neurotransmitter receptors. Fold change shows receptor expression of all neurotransmitter types in adult Lgr5^+^ cells. Differentially expressed genes were defined by a fold change ≥ 2 or ≤ −2, *p*-value < 0.05, and false discovery rate (FDR) < 0.05.

Glycine receptors in the adult murine retina are composed of two α and three β subunits [16]. Four different genes encode for different α subunits (α1–4) [17]. Lgr5^+^ cells expressed low levels of the β subunits gene Glrb (Figure 7; mean: 125). Furthermore, they expressed Glra2 (mean: 308) and Glra4 (mean: 382), which encode the α2 and α4 subunits, respectively.

In the retina, GABA is widely expressed by subpopulations of amacrine cells, bipolar cells, horizontal cells as well as retinal ganglion cells. Lgr5^+^ cells express a variety of GABA-receptors. The highest expressed is the GABA type A receptor subunit β3 (Gabrb3; mean: 1009). Moreover, both type B receptors are expressed (Gabbr1 and Gabbr2), as well as one type C receptor (Gabrr1).

Glutamate is the major excitatory neurotransmitter of the retina, expressed by photoreceptor cells, bipolar cells and retinal ganglion cells. Several glutamatergic receptors are expressed in Lgr5^+^ cells. Grik2 which encodes the ionotropic kainate receptor type subunit 2 (mean: 868) showed the highest signal values. Furthermore, Lgr5^+^ cells express receptors for acetylcholine (Chrnb2; mean: 338), dopamine (Drd1; mean: 803; Drd2; mean: 214), as well as serotonin (Htr1d; mean: 164).

## 3. Discussion

The statistical overrepresentation tests of differentially expressed genes of both developmental stages indicate that neither adult nor PN5 Lgr5^+^ cells harbor stem cell potential. None of the enriched GO terms of upregulated genes shows a positive association to NSC. On the contrary, downregulated genes in neonatal Lgr5^+^ cells compared to the negative population are enriched for genes associated to NSC maintenance and proliferation, indicating these processes prevail in the Lgr5^−^ cell population. Furthermore, several genes of GO terms related to the cell cycle are enriched in the neonatal Lgr5^−^ cell population. The heat maps depicting the expression values and fold changes of genes associated to NSC and cell cycle affirm these findings. Of the NSC markers analyzed only the NSC maintenance marker Prox1 is expressed. During embryonic retinogenesis Prox1-expression regulates cell cycle exit of progenitor cells [18]. However, it is also expressed in a subtype of horizontal cells and in AII amacrine cells. In both of these cell types Prox1-expression is upregulated during maturation indicating different functions of Prox1 during embryonic development and in adulthood. In addition, the expression values and fold changes of cell cycle genes clearly show that in neonates the expression of these genes is significantly upregulated in the Lgr5^−^ cell population compared to Lgr5^+^ cells. While Müller cells, bipolar cells, and rods are still being generated from proliferating retinal progenitors at PN5, glycinergic amacrine cells exit the cell cycle around postnatal day 4 [19,20]. This coincides with the appearance of Lgr5-expression suggesting that Lgr5 is expressed after glycinergic amacrine cell-fate specification.

Since Lgr5 is a Wnt target gene and acts as a stabilizer of canonical Wnt signaling different genes of this pathway were analyzed. The PANTHER analysis identified one GO term associated with Wnt signaling: “negative regulation of canonical Wnt signaling pathway”. These genes are enriched in the Lgr5^−^ cell population at PN5. However, from this result one cannot conclude that there is positive regulation of Wnt signaling in the Lgr5^+^ cell population. Indeed, the gene list analysis shows that Lgr5^+^ cells at PN5 express lower levels of Wnt antagonists/inhibitors. However, the genes show, overall, a low expression profile.

Regarding other members of the Wnt signaling cascade, none is significantly higher expressed in Lgr5^+^ cells compared to Lgr5^−^ cells at both developmental stages. Furthermore, Axin2 expression does not meet the signal threshold of 100. Axin2 is a transcriptional target of Wnt signaling and is generally regarded as an indicator of Wnt pathway activity [21]. These results suggest that even though Lgr5 is expressed, Wnt signaling is not active.

In this study, Lgr5^+^ cells of undamaged retinae were analyzed. Potentially injury to the retina might be necessary to induce stem cell properties of retinal Lgr5^+^ cells. In the liver a population of progenitors reside which only show regenerative capacity after acute injury, but do not contribute to homeostatic liver regeneration [22]. However, these cells only express Lgr5 after injury induction. That Lgr5 is expressed during homeostasis in retinal amacrine cells may suggest an alternative function in neurons than in stem cell populations.

In the murine retina, Lgr5 is expressed in glycinergic amacrine cells. However, this is not a homogenous cell population. Amacrine cells are the most diverse cell type of the retina and to date more than 40 different types have been described [23]. They are classified by their dendritic field (stratification and pattern), appearance of dendrites as well as neurotransmitter used. GABA and glycine are the two major neurotransmitters produced by amacrine cells [24]. Yet a small subpopulation remains for which the neurotransmitter is still unknown [13]. In addition to neurotransmitters, only a few molecular markers are known for the characterization of amacrine subpopulations. Recent advances in genetic profiling are changing this. For example, the efforts of Siegert et al. who generated a genetic address book of different retinal cell types [25]. By demonstrating that Lgr5^+^ cells display morphologies of different amacrine subpopulations, such as Flag A/B and AII cells, Sukhdeo et al. already suggested that the Lgr5^+^ amacrine cell population is heterogeneous [12].

Several receptors of the Lgr-family are alternatively spliced resulting in diverse transcript variants [26,27]. So far, three different splice variants of Lgr5 have been identified [8]. PCR was performed on adult mouse tissue to investigate whether Lgr5 is alternatively spliced in the retina (see Supplementary Data S1). Thereby, full-length transcript variants of variable sizes were found. However, these did not correspond to known variants Lgr5Δ5 or Lgr5Δ8 [28]. Osawa et al. demonstrated that Lgr5Δ5 expressing cells show increased proliferation [8]. As no proliferation is seen in adult retinal tissue, it is not surprising that this variant is not expressed in the retina. Additionally, heterogeneity in the expression of Lgr5 splice variants, e.g., fetal tissue-specific variants or injury/disease-related modifications in adult tissue could possibly diminish the ability to detect progenitor capability. Follow up studies would be necessary to investigate alternative variants in the retina. Since Lgr5 is composed of 18 exons, several different splice variants are possible.

## 4. Materials and Methods

### 4.1. Animals

Adult (6–8 week old) C57BL/6 mice of both sexes were obtained from the Central Animal Facility of the Department for BioMedical Research of the University of Bern, Bern, Switzerland. Lgr^5EGFP-Ires-CreERT2^ breeding male was a kind gift from Dr. Marta Roccio, Inner Ear Research Laboratory, Department of Otorhinolaryngology, Bern University Hospital, University of Bern, Bern, Switzerland. These transgenic mice express enhanced green fluorescent protein (EGFP) under the Lgr5 promoter and are bred on a C57BL/6 background. Animals were housed under standard temperature (22 ± 2 °C) and light/dark cycle (12/12 h) with food and water ad libitum. The animal experiments were approved by the Cantonal Veterinary Office in Bern, Switzerland (BE51/16, 21.08.2018).

### 4.2. Lgr5 Transcriptomics

For RNA isolation of FACS sorted cells, GFP^+^ retinae of PN5 and adult animals of Lgr5^EGFP-Ires-CreERT2^ knock-in mice were dissociated using papain (Worthington Biochemical, Freehold, NJ, USA). For that, 40 μL papain (10 mg/mL, 30 U/mg) was activated with 40 μL L-cysteine/EDTA mix (25 mM L-cysteine; 5 mM EDTA, both Sigma Aldrich, Buchs, Switzerland) in 320 μL HEPES buffer (100 mM) at 37 °C for 15 min. Extracted retinae were incubated in the activated papain solution at 37 °C for 7 min.

Tissue was washed by centrifugation with Hank’s Balanced Salt Solution (HBSS; Thermo Fisher Scientific, Waltham, MA, USA) + 2% fetal bovine serum (FBS) and gently triturated using a fire-polished Pasteur pipette. The filtered single cell solution was sorted according to their GFP fluorescence on a FACS Aria (Becton Dickinson, Allschwil, Switzerland) at the cytometry core facility of the Department for BioMedical Research (DBMR) of the Faculty of Medicine of the University of Bern. Both Lgr5^+^ and Lgr5^−^ cell fractions were collected in HBSS + 2% FBS.

Total RNA of FACS sorted cells was extracted using the PicoPure RNA isolation kit (Thermo Fisher Scientific) according to the manufacturer’s instructions. RNA concentration, as well as quality was assessed by an Experion RNA HighSens Chip (Bio-Rad, Cressier, Switzerland).

Microarray processing was performed by ATLAS Biolabs in Berlin, Germany. RNA samples were analyzed by Affymetrix Clariom S Mouse Array. Raw intensity values were normalized by Robust Multiarray Averaging (RMA) and analyzed with Transcriptome Analysis Console (TAC) software (Version 4; Thermo Fisher Scientific).

### 4.3. Analyses of DGE

The online platform PANTHER (http://www.pantherdb.org) was used to analyze the results of DGE [29]. Statistical overrepresentation test of GO biological processes was performed on differentially expressed genes. Thereby, enriched GO terms were identified within a target list of genes compared to a background list. As target list either up- or downregulated genes from DGE analysis were used. The background list consisted of all analyzed genes of the transcriptome array. Enrichment was calculated by the following formula:

$$\mathrm{Enrichment}\;=\;\frac{b/n}{B/N}$$

N = total number of genes

B = total number of genes associated with a specific GO term

n = the number of genes in the target set

b = the number of genes in the intersection

The *p*-values for enriched GO terms were calculated using Fisher’s exact test. Heat maps of signal values were generated in Excel.

## 5. Conclusions

In conclusion, the results of our transcriptome analysis suggest that Lgr5^+^ cells do not harbor stem cell properties. However, Lgr5^+^ cells are a heterogeneous population of amacrine subtypes. Possibly a small subtype of Lgr5^+^ cells do possess stem cell properties. Yet the employed techniques were not suitable in detecting them. Especially in the transcriptome analysis, the expression of stem cell markers in a small population may be masked. Therefore, single cell analysis to investigate such populations would have to be performed to further characterize properties of Lgr5^+^ cells in the retina.

## Figures and Tables

**Table 1:** Top 10 enriched GO terms of up- and downregulated genes in adult Lgr5^+^ compared to Lgr5^−^. DGE: Input from up (+) or downregulated (−) genes from differential gene expression; #GO: number of genes in the GO term; #Input: number of genes in the input list; Exp.: expected number of genes in the input list; FE: fold enrichment.

GO Term	DGE	#GO	#Input	Exp.	FE	*p* Value
Synaptic vesicle clustering	+	4	4	0.25	16.00	6.89 × 10^−4^
Postsynaptic density protein 95 clustering	+	6	5	0.38	13.33	2.40 × 10^−4^
Positive regulation of protein localization to synapse	+	5	4	0.31	12.80	1.18 × 10^−3^
Synaptic transmission, glycinergic	+	7	5	0.44	11.43	3.91 × 10^−4^
Neurotransmitter receptor transport, postsynaptic endosome to lysosome	+	7	5	0.44	11.43	3.91 × 10^−4^
Synaptic membrane adhesion	+	10	7	0.63	11.20	2.77 × 10^−5^
Neuron cell-cell adhesion	+	9	6	0.56	10.66	1.29 × 10^−4^
Postsynaptic neurotransmitter receptor diffusion trapping	+	9	6	0.56	10.66	1.29 × 10^−4^
Anterograde axonal protein transport	+	8	5	0.50	10.00	6.05 × 10^−4^
Negative regulation of protein kinase activity by regulation of protein phosphorylation	+	8	5	0.50	10.00	6.05 × 10^−4^
Phototransduction, visible light	−	9	5	0.40	12.52	1.99 × 10^−4^
Photoreceptor cell maintenance	−	40	20	1.77	11.27	2.48 × 10^−13^
Detection of light stimulus involved in sensory perception	−	20	10	0.89	11.27	2.52 × 10^−7^
Protein localization to non-motile cilium	−	12	6	0.53	11.27	6.91 × 10^−5^
Eye photoreceptor cell development	−	34	13	1.51	8.62	4.94 × 10^−8^
Camera-type eye photoreceptor cell differentiation	−	20	7	0.89	7.89	1.03 × 10^−4^
Non-motile cilium assembly	−	50	16	2.22	7.21	1.14 × 10^−8^
Phospholipid transport	−	60	12	2.66	4.51	4.69 × 10^−5^
Axoneme assembly	−	60	11	2.66	4.13	1.90 × 10^−4^
Smoothened signaling pathway	−	74	12	3.28	3.66	2.71 × 10^−4^

**Table 2:** Top 10 enriched GO terms of up- and downregulated genes in PN5 Lgr5^+^ cells compared to Lgr5^−^. DGE: Input from up (+) or downregulated (−) genes from differential gene expression; #GO: number of genes in the GO term; #Input: number of genes in the input list; Exp.: expected number of genes in the input list; FE: fold enrichment.

GO Term	DGE	#GO	#Input	Exp.	FE	*p* Value
Negative regulation of dopamine secretion	+	5	5	0.21	23.51	2.37 × 10^−5^
Spontaneous synaptic transmission	+	5	4	0.21	18.81	2.94 × 10^−4^
Postsynaptic neurotransmitter receptor diffusion trapping	+	9	6	0.38	15.67	1.65 × 10^−5^
Positive regulation of inhibitory postsynaptic potential	+	8	5	0.34	14.69	1.09 × 10^−4^
Postsynaptic density assembly	+	8	5	0.34	14.69	1.09 × 10^−4^
Trans-synaptic signaling, modulating synaptic transmission	+	7	4	0.30	13.44	7.21 × 10^−4^
Neurotransmitter receptor transport, postsynaptic endosome to lysosome	+	7	4	0.30	13.44	7.21 × 10^−4^
Positive regulation of AMPA receptor activity	+	9	5	0.38	13.06	1.64 × 10^−4^
Regulation of short-term neuronal synaptic plasticity	+	18	10	0.77	13.06	8.15 × 10^−8^
Regulation of synaptic vesicle priming	+	9	5	0.38	13.06	1.64 × 10^−4^
Retinal rod cell differentiation	−	4	4	0.17	24.18	1.52 × 10^−4^
Compartment pattern specification	−	4	3	0.17	18.14	1.94 × 10^−3^
Mitotic spindle midzone assembly	−	7	5	0.29	17.27	6.12 × 10^−5^
Negative regulation of photoreceptor cell differentiation	−	6	4	0.25	16.12	4.28 × 10^−4^
Regulation of attachment of spindle microtubules to kinetochore	−	12	7	0.50	14.11	5.03 × 10^−6^
Notch signaling involved in heart development	−	7	4	0.29	13.82	6.51 × 10^−4^
DNA unwinding involved in DNA replication	−	8	4	0.33	12.09	9.46 × 10^−4^
Blood vessel endothelial cell differentiation	−	8	4	0.33	12.09	9.46 × 10^−4^
Protein localization to kinetochore	−	13	6	0.54	11.16	6.70 × 10^−5^
Positive regulation of mammary gland epithelial cell proliferation	−	9	4	0.37	10.75	1.32 × 10^−3^

**Table 3:** Top 10 enriched GO terms of up- and downregulated genes in PN5 Lgr5^+^ cells compared to adult Lgr5^+^. DGE: Input from up (+) or downregulated (−) genes from differential gene expression; #GO: number of genes in the GO term; #Input: number of genes in the input list; Exp.: expected number of genes in the input list; FE: fold enrichment.

GO Term	DGE	#GO	#Input	Exp.	FE	*p* Value
Negative regulation of ubiquitin protein ligase activity	+	10	5	0.38	13.32	1.36 × 10^−4^
DNA damage response, signal transduction by p53 class mediator resulting in transcription of p21 class mediator	+	11	5	0.41	12.11	1.92 × 10^−4^
Ribosomal small subunit assembly	+	16	6	0.60	9.99	1.00 × 10^−4^
Regulation of development, heterochronic	+	15	5	0.56	8.88	6.04 × 10^−4^
Cytoplasmic translation	+	50	16	1.88	8.52	1.17 × 10^−9^
Ribosomal large subunit assembly	+	34	9	1.28	7.05	1.90 × 10^−5^
Sympathetic nervous system development	+	23	6	0.86	6.95	5.13 × 10^−4^
ATP-dependent chromatin remodeling	+	36	8	1.35	5.92	1.58 × 10^−4^
Positive regulation of Notch signaling pathway	+	40	8	1.50	5.33	2.97 × 10^−4^
Ventricular septum morphogenesis	+	44	8	1.65	4.84	5.21 × 10^−4^
Calcium ion export	−	10	5	0.52	9.61	5.82 × 10^−4^
Membrane repolarization	−	15	7	0.78	8.96	6.30 × 10^−5^
Cerebellar Purkinje cell differentiation	−	13	6	0.68	8.87	2.25 × 10^−4^
Sodium ion export across plasma membrane	−	11	5	0.57	8.73	8.12 × 10^−4^
Relaxation of muscle	−	18	7	0.94	7.47	1.56 × 10^−4^
G-protein coupled glutamate receptor signaling pathway	−	16	6	0.83	7.20	5.45 × 10^−4^
CGMP metabolic process	−	17	6	0.88	6.78	7.06 × 10^−4^
Long term synaptic depression	−	18	6	0.94	6.40	9.03 × 10^−4^
Neuron cellular homeostasis	−	18	6	0.94	6.40	9.03 × 10^−4^
Phototransduction	−	28	9	1.46	6.17	6.09 × 10^−5^

## Supplementary Materials

The following are available online at https://www.mdpi.com/1422-0067/20/14/3547/s1.

## Abbreviations

DGE	Differential gene expression
GO	Gene ontology
NSC	Neural stem cells
PCA	Principle component analysis
PANTHER	Protein ANalysis THrough Evolutionary Relationships
