# Supplementary material for: Transcriptome Analysis Did Not Show Endogenous Stem Cell Characteristics in Murine Lgr5+ Retinal Cells
Source: Int J Mol Sci. 2019 Jul 19;20(14):3547. doi: 10.3390/ijms20143547 (PMC6678859; doi:10.3390/ijms20143547)
Supplement: Supplementary file 1 [file ijms-20-03547-s001.pdf]

SUPPORTING INFORMATION FOR:

Transcriptome analysis did not show endogenous stem cell characteristics in murine Lgr5$^+$ retinal cells

Carolyn Trepp [1,2], Ana Maria Quintela Pousa [2,3] and Volker Enzmann [2,3]*

[1] F. Hoffmann-La Roche, Basel, Switzerland; carolyn.trepp@roche.com
[2] Department of Ophthalmology, University Hospital of Bern, University of Bern, Bern, Switzerland; anamaria.quintelapousa@extern.insel.ch
[3] Department for BioMedical Research, University of Bern, Bern, Switzerland
* Correspondence: volker.enzmann@insel.ch

Table of content

Lgr5 splice variant analysis	S1
Electropherograms and virtual gels of RNA samples from Experion analysis.	Figure S1
Quality assessment of microarray analysis.	Figure S2
Principal component analysis of the eight-transcriptome profiles.	Figure S3
Heatmap of gene expression levels and fold changes of NSC maintenance markers.	Figure S4
RNA sample information.	Table S1
Full list of overrepresented GO terms of upregulated genes in adult Lgr5$^+$ compared to adult Lgr5$^-$.	Table S2
Full list of overrepresented GO terms of downregulated genes in adult Lgr5$^+$ compared to adult Lgr5$^-$.	Table S3
Full list of overrepresented GO terms of upregulated genes in PN5 Lgr5$^+$ compared to PN5 Lgr5$^-$.	Table S4
Full list of overrepresented GO terms of downregulated genes in PN5 Lgr5$^+$ compared to adult Lgr5$^-$.	Table S5
Full list of overrepresented GO terms of upregulated genes in PN5 Lgr5$^+$ compared to adult Lgr5$^+$.	Table S6
Full list of overrepresented GO terms of downregulated genes in PN5 Lgr5$^+$ compared to adult Lgr5$^+$.	Table S7

Supplementary Data 1: Lgr5 splice variant analysis

To investigate whether Lgr5 is alternatively spliced in the mouse retina, RT-PCR was performed. As positive control (PC) mouse total RNA (XpressRef Universal Total RNA), which is prepared from a wide range of organs, was used. RNA was isolated from mouse retinae using the RNeasy Micro Kit according to the manufacturer's instructions and its quality was analyzed by Experion HighSens Chip. Reverse transcription was performed using the iScript Reverse Transcription Kit. Mouse retinae were examined for the specific deletion of exon 5 (Lgr5Δ5) or exon 8 (Lgr5Δ8), or deletion of various exons between exon 1 and 13 (Lgr5Δ1-13). All primers were designed using the online primer design tool (https://www.ncbi.nlm.nih.gov/tools/primer-blast/). The primers were analyzed for possible hairpin and primer-dimer formation by the OligoAnalyzer online tool (https://eu.idtdna.com/calc/analyzer). Appropriate primers were synthesized by Microsynth AG, Balgach, Switzerland:

Species	Primer	Forward and reverse sequence	Amplicon [bp]
Mouse	Lgr5e5	CAGAACAACCAGCTGAGACAG	341 (FL)
		CTGTGGAGTCCATCAAAGCAT	125 (Δ5)
	Lgr5e8	CACTGCAATCAAGACACTCTC	132 (FL)
		TGGATGGGGTTGTCATAGAAGT	60 (Δ8)
	Lgr5e1-13	AACCTCAGCGTCTTCACCTC	1006 (FL)
		ACAACTGCTGAAAAGTGCTGC	790 (Δ5)
			934 (Δ8)

GoTaq® G2 DNA Polymerase kit (Promega, Amriswil, Switzerland) was used according to manufacturer's instructions. PCR products were separated on a 2% agarose gel in 0.5% TBE buffer and bands were visualized using a Gel Doc EZ Imager (Bio-Rad).

In a first step, primers amplifying exons 1-13, which encode the leucine-rich repeats, were employed. Thereby, several bands were seen in mouse retina and the positive controls (PC; Figure S1-1). In both mouse retina and PC besides the full-length amplicon at 1006 bp (FL) a second band was detected at 790 bp which may represent the splice variant Lgr5Δ5. The strong Lgr5FL band from mouse retina may mask Lgr5Δ8 (934 bp).

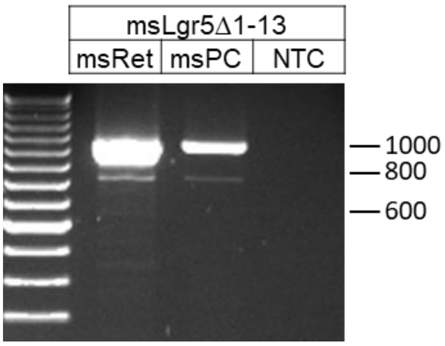

Figure S1-1: PCR for the detection of Lgr5 splice variants in the mouse retina. All samples showed a clear band around 1'000 bp representing the full-length Lgr5 amplicon (Lgr5FL). Several other weaker bands were detected, which may represent different splice variants. msRet: mouse retina, msPC: mouse positive control, NTC: non-template control.

To verify the amplicons seen as weak bands with primers amplifying exons 1-13, specific primers were designed to detect potential deletions of exon 5 and 8. Only one amplicon per primer pair was detected (Figure S1-2). These showed the predicted size of Lgr5FL amplicons at 341 bp and 132 for Lgr5e5 and Lgr5e8, respectively. Hence this second band does not correspond to either variant Lgr5Δ5 (125 bp) or Lgr5Δ8 (60 bp). Follow up studies would be

necessary to investigate alternative variants in the retina. Since Lgr5 is composed of 18 exons, several different splice variants are possible.

Fetal, PN5 and injured retinae were not analyzed for splice variants.

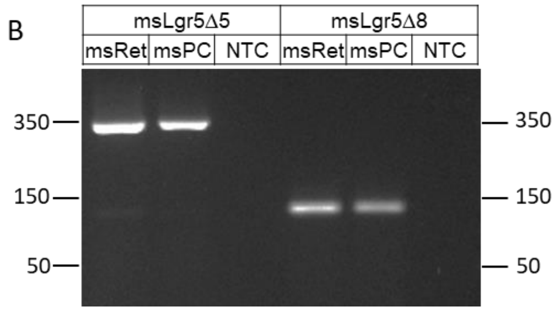

Figure S1-2: PCR for the detection of Lgr5Δ5 and Lgr5Δ8 splice variants in mouse retinal tissue. Retinal tissue of mouse only exhibited bands for the full-length Lgr5 amplicons (Lgr5FL). msRet: mouse retina, msPC: mouse positive control, NTC: non-template control.

Supplementary Figure S1. Electropherograms and virtual gels of RNA samples from Experion analysis. All samples show two distinct 18S and 28S peaks in the electropherogram and bands in the virtual gels. Peak at 25s represents the lower marker for correct alignment.

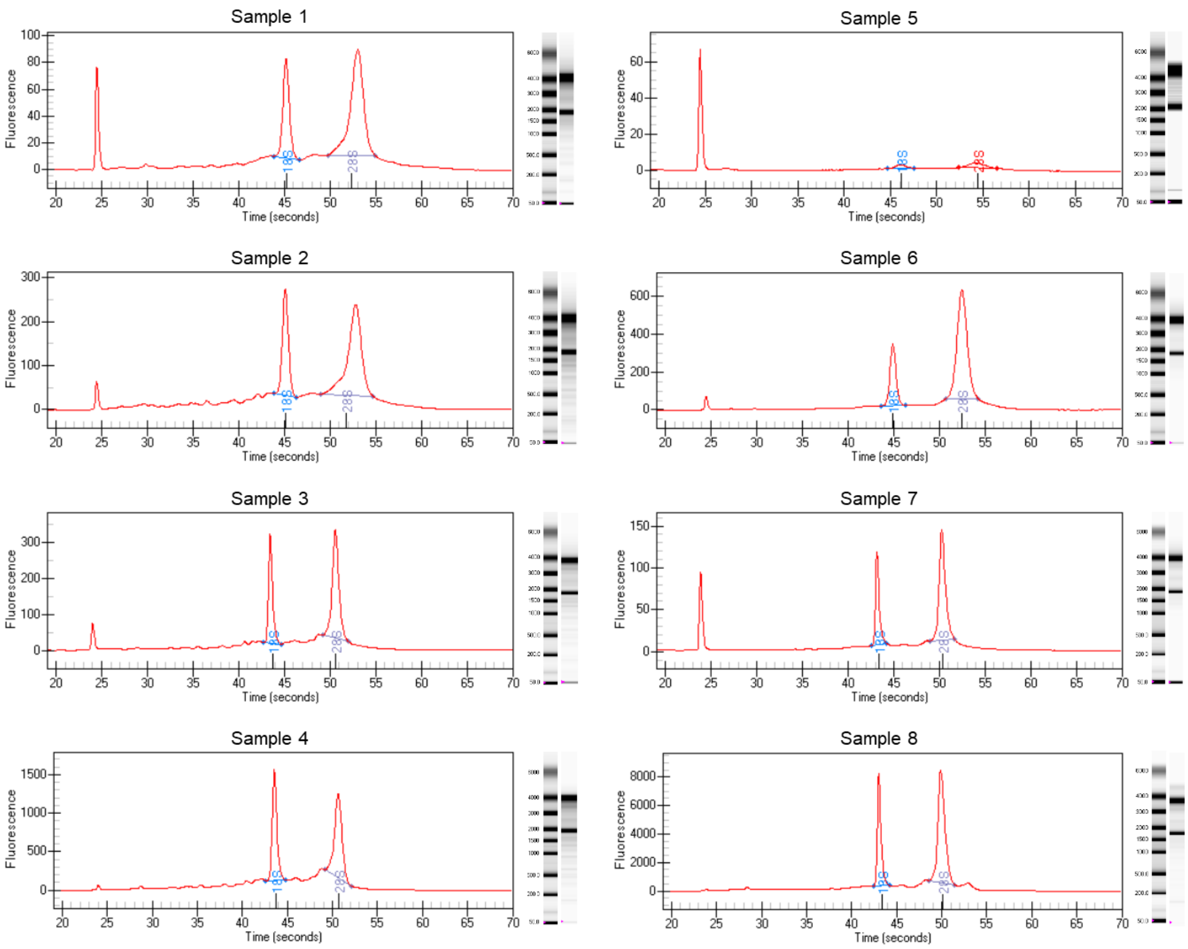

Supplementary Figure S2. Quality assessment of microarray analysis. All samples showed increasing signal values of hybridization controls (A), consistent AUC values (B) and similar distributions of signal values (C).

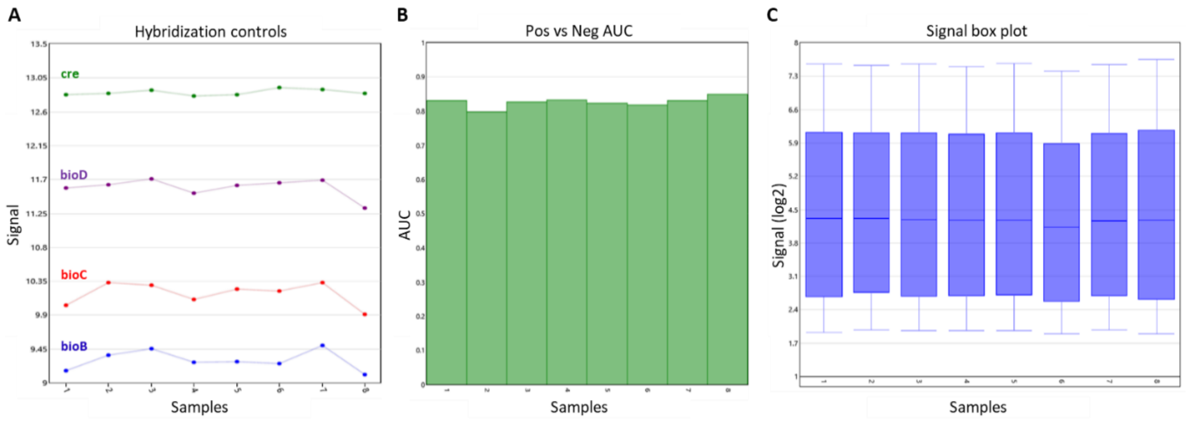

Supplementary Figure S3. Principal component analysis of the eight-transcriptome profiles. Samples clustered according to their developmental stage and Lgr5-expression.

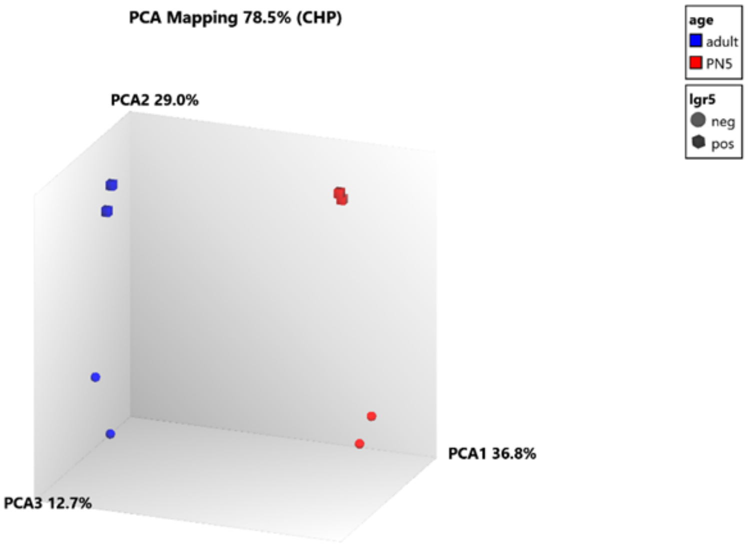

Supplementary Figure S4. Heatmap of gene expression levels and fold changes of NSC maintenance markers. Prox1 is upregulated in both PN5 and adult Lgr5+ cells compared to the negative cell population of the respective developmental stage.

	PN5							Adult							Pos: Pn5 vs. Adult		
	Lgr5+ PN5 1	Lgr5+ PN5 2	Lgr5- PN5 1	Lgr5- PN5 2	Fold Change	P-val	FDR P-val	Lgr5+ Adult 1	Lgr5+ Adult 2	Lgr5- Adult 1	Lgr5- Adult 2	Fold Change	P-val	FDR P-val	Fold Change	P-val	FDR P-val
Aspm	[illegible]	[illegible]	27	74	-7.18	6.4E-05	2.4E-03	[illegible]	[illegible]	[illegible]	[illegible]	-1.20	5.3E-01	8.0E-01	1.48	2.9E-02	1.4E-01
Cdh2	613	506	1706	1651	-3.01	6.6E-05	2.5E-03	424	437	315	350	1.30	9.7E-02	3.3E-01	1.29	9.0E-02	2.9E-01
Dll1	[illegible]	[illegible]	79	124	-12.97	6.8E-07	2.0E-04	[illegible]	[illegible]	[illegible]	[illegible]	-1.54	6.9E-02	2.7E-01	1.61	3.4E-02	1.5E-01
Fancc	15	15	15	19	-1.13	4.7E-01	7.4E-01	21	21	17	19	1.17	3.0E-01	6.2E-01	-1.40	2.7E-02	1.3E-01
Fancd2	[illegible]	[illegible]	[illegible]	12	-1.28	2.0E-01	4.9E-01	[illegible]	[illegible]	[illegible]	[illegible]	-1.12	5.4E-01	8.0E-01	1.10	5.0E-01	7.4E-01
Foxo1	14	18	28	35	-1.92	3.7E-03	4.4E-02	24	20	59	42	-2.28	7.0E-04	1.0E-02	-1.35	6.3E-02	2.3E-01
Foxo3	21	19	26	20	-1.14	4.7E-01	7.3E-01	25	23	21	25	1.03	8.5E-01	9.5E-01	-1.20	1.9E-01	4.5E-01
Foxo3	21	19	26	20	-1.14	4.7E-01	7.3E-01	25	23	21	25	1.03	8.5E-01	9.5E-01	-1.20	1.9E-01	4.5E-01
Fut10	16	21	42	61	-2.88	4.0E-04	9.1E-03	19	12	43	51	-3.16	3.0E-04	4.8E-03	1.19	4.0E-01	6.6E-01
Hes1	27	28	27	31	-1.07	6.8E-01	8.6E-01	26	19	39	24	-1.36	1.3E-01	4.0E-01	1.21	2.4E-01	5.1E-01
Hes1	27	28	27	31	-1.07	6.8E-01	8.6E-01	26	19	39	24	-1.36	1.3E-01	4.0E-01	1.21	2.4E-01	5.1E-01
Hes5	100	113	117	153	-1.26	2.0E-01	4.9E-01	105	86	129	97	-1.18	3.4E-01	6.6E-01	1.12	4.3E-01	6.9E-01
Hes5	100	113	117	153	-1.26	2.0E-01	4.9E-01	105	86	129	97	-1.18	3.4E-01	6.6E-01	1.12	4.3E-01	6.9E-01
Hook3	97	77	76	63	1.25	2.1E-01	5.1E-01	63	48	133	85	-1.93	6.1E-03	5.0E-02	1.57	1.9E-02	1.1E-01
Igf2bp1	[illegible]	15	11	16	-1.23	4.1E-01	6.9E-01	[illegible]	[illegible]	[illegible]	[illegible]	1.06	8.0E-01	9.4E-01	2.54	3.5E-03	3.3E-02
Mapk8	50	48	60	48	-1.10	5.8E-01	8.0E-01	48	42	64	52	-1.27	1.4E-01	4.2E-01	1.09	5.4E-01	7.7E-01
Mcph1	76	66	66	90	-1.09	6.2E-01	8.3E-01	55	57	25	29	2.09	9.0E-04	1.2E-02	1.26	1.1E-01	3.3E-01
Mmp24	56	72	16	36	2.47	3.0E-03	3.8E-02	68	96	39	47	1.90	1.7E-02	1.0E-01	-1.27	1.8E-01	4.3E-01
Pcm1	203	192	205	184	1.02	9.2E-01	9.7E-01	157	173	144	151	1.12	4.3E-01	7.3E-01	1.20	1.9E-01	4.5E-01
Prox1	1156	1119	421	308	3.16	7.7E-05	2.7E-03	828	949	112	474	3.85	3.6E-03	3.4E-02	1.28	8.8E-02	2.8E-01
Prrx1	[illegible]	[illegible]	[illegible]	[illegible]	-1.27	3.2E-01	6.1E-01	[illegible]	[illegible]	[illegible]	[illegible]	-1.07	8.0E-01	9.3E-01	1.00	9.9E-01	1.0E+00
Sct	10	14	24	29	-2.21	1.6E-03	2.4E-02	16	17	15	10	1.33	1.4E-01	4.2E-01	-1.40	5.8E-02	2.2E-01
Sox2	34	27	346	387	-11.96	9.2E-08	7.1E-05	24	[illegible]	29	24	-1.83	4.3E-02	2.0E-01	2.09	3.0E-02	1.4E-01
Srrt	224	260	297	274	-1.18	3.2E-01	6.1E-01	176	230	175	201	1.07	6.6E-01	8.7E-01	1.20	2.5E-01	5.2E-01
Ss18	139	153	155	149	-1.04	8.0E-01	9.2E-01	122	130	118	101	1.16	3.3E-01	6.5E-01	1.16	2.8E-01	5.5E-01
Wdr62	10	[illegible]	[illegible]	12	-1.14	5.0E-01	7.5E-01	[illegible]	10	[illegible]	[illegible]	1.38	9.0E-02	3.2E-01	-1.09	5.6E-01	7.8E-01

Supplementary Table S1. RNA sample information

Sample	Age	Lgr5+/-	# cells	RNA [ng/µl]	total RNA [ng]	RQI
1	Adult	+	149'013	5.40	48.60	9.7
2	Adult	-	1'000'000	17.37	156.33	9.1
3	Adult	+	138'975	9.18	100.98	9.4
4	Adult	-	1'000'000	45.65	502.15	9.1
5	PN5	+	64'230	0.51	4.27	-
6	PN5	-	1'000'000	21.40	177.62	9.9
7	PN5	+	58'910	3.70	40.70	10.0
8	PN5	-	1'000'000	23.90	262.90	9.8

Supplementary Table S2. Full list of overrepresented GO terms of upregulated genes in adult Lgr5$^+$ compared to adult Lgr5$^-$. #GO: number of genes in GO term; #Input: number of genes in input list; Exp.: expected number of genes in input list; FE: fold enrichment.

GO term	#GO	#Input	Exp.	FE	P value
Synaptic vesicle clustering	4	4	0.25	16	6.9E-04
Postsynaptic density protein 95 clustering	6	5	0.38	13.33	2.4E-04
Positive regulation of protein localization to synapse	5	4	0.31	12.8	1.2E-03
Synaptic transmission, glycinergic	7	5	0.44	11.43	3.9E-04
Neurotransmitter receptor transport, postsynaptic endosome lysosome	7	5	0.44	11.43	3.9E-04 to
Synaptic membrane adhesion	10	7	0.63	11.2	2.8E-05
Neuron cell-cell adhesion	9	6	0.56	10.66	1.3E-04
Postsynaptic neurotransmitter receptor diffusion trapping	9	6	0.56	10.66	1.3E-04
Anterograde axonal protein transport	8	5	0.5	10	6.1E-04
Negative regulation of protein kinase activity by regulation of protein phosphorylation	8	5	0.5	10	6.1E-04
Gamma-aminobutyric acid transport	8	5	0.5	10	6.1E-04
Non-associative learning	8	5	0.5	10	6.1E-04
Cerebellar granule cell differentiation	10	6	0.63	9.6	2.0E-04
Positive regulation of AMPA receptor activity	9	5	0.56	8.89	9.0E-04
Postsynaptic membrane assembly	9	5	0.56	8.89	9.0E-04
Excitatory synapse assembly	11	6	0.69	8.73	2.9E-04
Presynaptic membrane organization	10	5	0.63	8	1.3E-03
Cerebellar Purkinje cell differentiation	13	6	0.81	7.38	5.7E-04
Short-term memory	13	6	0.81	7.38	5.7E-04
Regulation of short-term neuronal synaptic plasticity	18	8	1.13	7.11	8.5E-05
Detection of mechanical stimulus involved in sensory perception of pain	19	8	1.19	6.74	1.1E-04
Vocalization behavior	15	6	0.94	6.4	1.0E-03
Regulation of vascular endothelial growth factor signaling pathway	15	6	0.94	6.4	1.0E-03
Membrane repolarization	15	6	0.94	6.4	1.0E-03
Positive regulation of synaptic transmission, glutamatergic	36	14	2.25	6.22	7.0E-07
Regulation of presynapse organization	16	6	1	6	1.4E-03
Regulation of long term synaptic depression	16	6	1	6	1.4E-03
Excitatory postsynaptic potential	38	14	2.38	5.89	1.2E-06
Positive regulation of synapse assembly	73	26	4.56	5.7	6.5E-11
Positive regulation of cellular component organization	1198	129	74.89	1.72	1.2E-08
Regulation of synapse assembly	94	30	5.88	5.11	2.1E-11
Gamma-aminobutyric acid signaling pathway	27	9	1.69	5.33	1.8E-04
Branching involved in mammary gland duct morphogenesis	26	8	1.63	4.92	6.5E-04
Negative regulation of synapse organization	23	7	1.44	4.87	1.5E-03
Neuronal action potential	34	10	2.13	4.7	1.9E-04
Positive regulation of excitatory postsynaptic potential	34	10	2.13	4.7	1.9E-04
Regulation of neuron migration	48	14	3	4.67	1.1E-05
Startle response	31	9	1.94	4.64	4.3E-04
Regulation of long-term neuronal synaptic plasticity	35	10	2.19	4.57	2.3E-04
Calcium-dependent cell-cell adhesion via plasma membrane adhesion molecules	35	10	2.19	4.57	2.3E-04 cell
Cardiac muscle cell action potential involved in contraction	28	8	1.75	4.57	9.7E-04
Cardiac muscle cell contraction	30	8	1.88	4.27	1.4E-03
Cyclic nucleotide metabolic process	32	9	2	4.5	5.2E-04
Mating behavior	32	9	2	4.5	5.2E-04
Regulation of long-term synaptic potentiation	43	12	2.69	4.46	6.8E-05
Adult walking behavior	44	12	2.75	4.36	8.2E-05
Receptor clustering	52	14	3.25	4.31	2.4E-05
Semaphorin-plexin signaling pathway	38	10	2.38	4.21	4.0E-04
Neuron recognition	46	12	2.88	4.17	1.2E-04

Long-term synaptic potentiation	54	14	3.38	4.15	3.4E-05
Homophilic cell adhesion via plasma membrane adhesion molecules	102	26	6.38	4.08	2.4E-08
Sodium ion transmembrane transport	55	14	3.44	4.07	4.1E-05
Negative regulation of synaptic transmission	67	17	4.19	4.06	6.5E-06
Regulation of voltage-gated calcium channel activity	36	9	2.25	4	1.1E-03
Synaptic transmission, glutamatergic	36	9	2.25	4	1.1E-03
Synaptic vesicle exocytosis	56	14	3.5	4	4.8E-05
Membrane depolarization	45	11	2.81	3.91	3.6E-04
Regulation of neurotransmitter secretion	70	17	4.38	3.88	1.1E-05
Social behavior	50	12	3.13	3.84	2.3E-04
Response to cocaine	42	10	2.63	3.81	7.9E-04
Dendrite morphogenesis	70	16	4.38	3.66	3.6E-05
Neuromuscular process controlling balance	66	15	4.13	3.64	6.7E-05
Positive regulation of protein localization to plasma membrane	53	12	3.31	3.62	3.6E-04
Regulation of dopamine secretion	45	10	2.81	3.55	1.2E-03
Neuron projection extension	63	14	3.94	3.55	1.4E-04
Fear response	45	10	2.81	3.55	1.2E-03
Receptor internalization	60	13	3.75	3.47	3.0E-04
Phosphatidylinositol-mediated signaling	57	12	3.56	3.37	6.4E-04
Regulation of postsynapse organization	72	15	4.5	3.33	1.6E-04
Cerebral cortex cell migration	54	11	3.38	3.26	1.3E-03
Potassium ion transmembrane transport	130	26	8.13	3.2	1.4E-06
Calcium ion transmembrane transport	135	27	8.44	3.2	8.8E-07
Regulation of synaptic vesicle cycle	55	11	3.44	3.2	1.5E-03
Regulation of potassium ion transmembrane transporter activity	55	11	3.44	3.2	1.5E-03
Regulation of calcium ion-dependent exocytosis	60	12	3.75	3.2	9.4E-04
Negative regulation of epithelial cell migration	66	13	4.13	3.15	6.7E-04
Protein localization to plasma membrane	164	32	10.25	3.12	1.5E-07
Regulation of cAMP-mediated signaling	62	12	3.88	3.1	1.2E-03
Regulation of dendrite morphogenesis	109	21	6.81	3.08	2.3E-05
Regulation of G-protein coupled receptor protein signaling pathway	123	23	7.69	2.99	1.5E-05
Positive regulation of dendrite development	102	19	6.38	2.98	8.2E-05
Negative regulation of ERK1 and ERK2 cascade	71	13	4.44	2.93	1.2E-03
Negative regulation of cell morphogenesis involved differentiation	99	18	6.19	2.91	1.6E-04 in
Positive regulation of axonogenesis	96	17	6	2.83	3.2E-04
Neutral lipid metabolic process	86	15	5.38	2.79	8.1E-04
Neuron migration	138	24	8.63	2.78	2.7E-05
Axon guidance	193	33	12.07	2.74	1.3E-06
Associative learning	100	17	6.25	2.72	4.9E-04
Regulation of circadian rhythm	105	17	6.56	2.59	8.0E-04
Transmembrane receptor protein tyrosine kinase signaling pathway	320	51	20	2.55	1.8E-08
Regulation of extent of cell growth	121	19	7.56	2.51	7.6E-04
Glial cell differentiation	162	23	10.13	2.27	7.3E-04
Cyclic-nucleotide-mediated signaling	155	22	9.69	2.27	9.9E-04
Negative regulation of neuron projection development	167	23	10.44	2.2	9.4E-04
Regulation of heart contraction	167	23	10.44	2.2	9.4E-04
Establishment or maintenance of cell polarity	183	24	11.44	2.1	1.5E-03
Protein autophosphorylation	193	25	12.07	2.07	1.3E-03
Regulation of peptide hormone secretion	216	27	13.5	2	1.4E-03
Negative regulation of secretion by cell	217	27	13.57	1.99	1.5E-03
Regulation of GTPase activity	289	35	18.07	1.94	4.9E-04
Response to toxic substance	284	33	17.75	1.86	1.6E-03
Actin cytoskeleton organization	442	51	27.63	1.85	9.2E-05
Cellular response to organonitrogen compound	379	43	23.69	1.81	4.9E-04
Cellular response to hormone stimulus	400	43	25.01	1.72	1.4E-03
Positive regulation of cell migration	509	52	31.82	1.63	1.4E-03

Cellular response to oxygen-containing compound	784	78	49.01	1.59	1.5E-04
Chemical homeostasis	1017	96	63.58	1.51	1.8E-04
Positive regulation of protein phosphorylation	968	88	60.51	1.45	9.8E-04

Supplementary Table S3. Full list of overrepresented GO terms of downregulated genes in adult Lgr5+ compared to adult Lgr5-. #GO: number of genes in GO term; #Input: number of genes in input list; Exp.: expected number of genes in input list; FE: fold enrichment.

GO term	#GO	#Input	Exp.	FE	P value
Phototransduction, visible light	9	5	0.4	12.52	2.0E-04
Photoreceptor cell maintenance	40	20	1.77	11.27	2.5E-13
Detection of light stimulus involved in sensory perception	20	10	0.89	11.27	2.5E-07
Protein localization to non-motile cilium	12	6	0.53	11.27	6.9E-05
Eye photoreceptor cell development	34	13	1.51	8.62	4.9E-08
Camera-type eye photoreceptor cell differentiation	20	7	0.89	7.89	1.0E-04
Non-motile cilium assembly	50	16	2.22	7.21	1.1E-08
Phospholipid transport	60	12	2.66	4.51	4.7E-05
Axoneme assembly	60	11	2.66	4.13	1.9E-04
Smoothened signaling pathway	74	12	3.28	3.66	2.7E-04
Microtubule-based movement	232	28	10.29	2.72	6.4E-06
Organophosphate biosynthetic process	373	34	16.55	2.05	1.7E-04
Phosphate-containing compound metabolic process	1661	107	73.68	1.45	2.1E-04
Regulation of localization	2625	156	116.44	1.34	2.2E-04
Positive regulation of biological process	5520	302	244.86	1.23	4.3E-05

Supplementary Table S4. Full list of overrepresented GO terms of upregulated genes in PN5 Lgr5+ compared to PN5 Lgr5-. #GO: number of genes in GO term; #Input: number of genes in input list; Exp.: expected number of genes in input list; FE: fold enrichment.

GO term	#GO	#Input	Exp.	FE	P value
Negative regulation of dopamine secretion	5	5	0.21	23.51	2.4E-05
Spontaneous synaptic transmission	5	4	0.21	18.81	2.9E-04
Postsynaptic neurotransmitter receptor diffusion trapping	9	6	0.38	15.67	1.7E-05
Positive regulation of inhibitory postsynaptic potential	8	5	0.34	14.69	1.1E-04
Postsynaptic density assembly	8	5	0.34	14.69	1.1E-04
Trans-synaptic signaling, modulating synaptic transmission	7	4	0.3	13.44	7.2E-04
Neurotransmitter receptor transport, postsynaptic endosome lysosome	7	4	0.3	13.44	7.2E-04 to
Positive regulation of AMPA receptor activity	9	5	0.38	13.06	1.6E-04
Regulation of short-term neuronal synaptic plasticity	18	10	0.77	13.06	8.2E-08
Regulation of synaptic vesicle priming	9	5	0.38	13.06	1.6E-04
Cellular response to histamine	8	4	0.34	11.76	1.1E-03
Protein localization to axon	8	4	0.34	11.76	1.1E-03
Neurotransmitter receptor internalization	12	6	0.51	11.76	5.5E-05
Glycine transport	10	5	0.43	11.76	2.4E-04
Non-associative learning	8	4	0.34	11.76	1.1E-03
SA node cell action potential	8	4	0.34	11.76	1.1E-03
Synaptic transmission, GABAergic	19	9	0.81	11.14	1.0E-06
Vocalization behavior	15	7	0.64	10.97	1.8E-05
Neuronal action potential propagation	11	5	0.47	10.69	3.4E-04
Regulation of gamma-aminobutyric acid secretion	11	5	0.47	10.69	3.4E-04
Synaptic vesicle maturation	11	5	0.47	10.69	3.4E-04
Neurotransmitter-gated ion channel clustering	11	5	0.47	10.69	3.4E-04
Gamma-aminobutyric acid signaling pathway	27	12	1.15	10.45	2.8E-08
Acetylcholine receptor signaling pathway	21	9	0.89	10.08	2.0E-06
Negative regulation of adenylate cyclase activity	12	5	0.51	9.8	4.6E-04
Positive regulation of glutamate secretion	15	6	0.64	9.4	1.5E-04

Positive regulation of synaptic transmission, glutamatergic	36	14	1.53	9.14	7.6E-09
Calcium ion-regulated exocytosis of neurotransmitter	16	6	0.68	8.82	1.9E-04
Positive regulation of neurotransmitter secretion	20	7	0.85	8.23	8.0E-05
Synaptic transmission, cholinergic	21	7	0.89	7.84	1.0E-04
Locomotory exploration behavior	15	5	0.64	7.84	1.0E-03
Positive regulation of calcium ion-dependent exocytosis	23	7	0.98	7.16	1.6E-04
Regulation of dopamine metabolic process	20	6	0.85	7.05	5.2E-04
Protein localization to synapse	44	13	1.87	6.95	3.7E-07
Positive regulation of excitatory postsynaptic potential	34	10	1.45	6.92	8.5E-06
Cell-cell adhesion mediated by cadherin	24	7	1.02	6.86	2.1E-04
Positive regulation of long-term synaptic potentiation	24	7	1.02	6.86	2.1E-04
Response to nicotine	32	9	1.36	6.61	3.2E-05
Synaptic transmission, glutamatergic	36	10	1.53	6.53	1.3E-05
Postsynaptic membrane organization	27	7	1.15	6.1	3.8E-04
Neuromuscular synaptic transmission	31	8	1.32	6.07	1.5E-04
Regulation of long-term neuronal synaptic plasticity	35	9	1.49	6.05	5.9E-05
Calcium-dependent cell-cell adhesion via plasma membrane cell adhesion molecules	35	9	1.49	6.05	5.9E-05
Chloride transmembrane transport	39	10	1.66	6.03	2.3E-05
Membrane depolarization during action potential	24	6	1.02	5.88	1.2E-03
Regulation of dendrite extension	28	7	1.19	5.88	4.5E-04
Long-term memory	37	9	1.57	5.72	8.5E-05
Synaptic vesicle endocytosis	29	7	1.23	5.68	5.4E-04
Long-term synaptic potentiation	54	13	2.3	5.66	2.6E-06
Regulation of postsynaptic membrane potential	59	14	2.51	5.58	1.3E-06
Vesicle docking involved in exocytosis	38	9	1.62	5.57	1.0E-04
Neuronal action potential	34	8	1.45	5.53	2.6E-04
Startle response	31	7	1.32	5.31	7.7E-04
Behavioral fear response	41	9	1.74	5.16	1.7E-04
Glutamate receptor signaling pathway	37	8	1.57	5.08	4.2E-04
Homophilic cell adhesion via plasma membrane adhesion molecules	102	22	4.34	5.07	5.6E-09
Negative regulation of synaptic transmission	67	14	2.85	4.91	4.6E-06
Dopamine receptor signaling pathway	34	7	1.45	4.84	1.2E-03
Positive regulation of synapse assembly	73	15	3.1	4.83	2.5E-06
Smooth muscle contraction	50	10	2.13	4.7	1.4E-04
Chemical synaptic transmission, postsynaptic	45	9	1.91	4.7	3.1E-04
Social behavior	50	10	2.13	4.7	1.4E-04
Neuron recognition	46	9	1.96	4.6	3.5E-04
Neuromuscular junction development	47	9	2	4.5	4.1E-04
Axonal transport	48	9	2.04	4.41	4.7E-04
Adult walking behavior	44	8	1.87	4.27	1.1E-03
Regulation of postsynapse organization	72	13	3.06	4.25	3.9E-05
Sensory perception of pain	100	18	4.25	4.23	1.4E-06
Neuromuscular process controlling balance	66	11	2.81	3.92	2.8E-04
Visual learning	66	11	2.81	3.92	2.8E-04
Regulation of dendrite morphogenesis	109	18	4.64	3.88	4.1E-06
Response to anesthetic	56	9	2.38	3.78	1.2E-03
Negative regulation of calcium ion transport	56	9	2.38	3.78	1.2E-03
Dendrite morphogenesis	70	11	2.98	3.69	4.4E-04
Response to ethanol	67	10	2.85	3.51	1.1E-03
Negative regulation of axonogenesis	67	10	2.85	3.51	1.1E-03
Positive regulation of axonogenesis	96	14	4.08	3.43	1.5E-04
Regulation of calcium ion transmembrane transporter activity	76	11	3.23	3.4	8.1E-04
Calcium ion transmembrane transport	135	17	5.74	2.96	1.6E-04
Regulation of extent of cell growth	121	15	5.15	2.91	4.5E-04
Adenylate cyclase-modulating G-protein coupled receptor signaling pathway	188	23	8	2.88	1.9E-05
Potassium ion transmembrane transport	130	15	5.53	2.71	8.8E-04
Negative regulation of neuron apoptotic process	166	19	7.06	2.69	2.1E-04

Axon guidance	193	22	8.21	2.68	7.3E-05
Protein localization to plasma membrane	164	18	6.98	2.58	4.9E-04
Transmembrane receptor protein tyrosine kinase signaling pathway	320	29	13.61	2.13	3.0E-04
Brain development	590	46	25.09	1.83	2.1E-04
Cytoskeleton organization	977	70	41.55	1.68	5.4E-05

Supplementary Table S5. Full list of overrepresented GO terms of downregulated genes in PN5 Lgr5$^+$ compared to adult Lgr5$^-$. #GO: number of genes in GO term; #Input: number of genes in input list; Exp.: expected number of genes in input list; FE: fold enrichment.

GO term	#GO	#Input	Exp.	FE	P value
Retinal rod cell differentiation	4	4	0.17	24.18	1.5E-04
Compartment pattern specification	4	3	0.17	18.14	1.9E-03
Mitotic spindle midzone assembly	7	5	0.29	17.27	6.1E-05
Negative regulation of photoreceptor cell differentiation	6	4	0.25	16.12	4.3E-04
Regulation of attachment of spindle microtubules to kinetochore	12	7	0.5	14.11	5.0E-06
Notch signaling involved in heart development	7	4	0.29	13.82	6.5E-04
DNA unwinding involved in DNA replication	8	4	0.33	12.09	9.5E-04
Blood vessel endothelial cell differentiation	8	4	0.33	12.09	9.5E-04
Protein localization to kinetochore	13	6	0.54	11.16	6.7E-05
Positive regulation of mammary gland epithelial cell proliferation	9	4	0.37	10.75	1.3E-03
Positive regulation of integrin-mediated signaling pathway	9	4	0.37	10.75	1.3E-03
Glomerular basement membrane development	9	4	0.37	10.75	1.3E-03
Cellular response to follicle-stimulating hormone stimulus	9	4	0.37	10.75	1.3E-03
Mitotic chromosome condensation	14	6	0.58	10.36	9.3E-05
Left/right axis specification	12	5	0.5	10.08	4.1E-04
Marginal zone B cell differentiation	10	4	0.41	9.67	1.8E-03
Regulation of somitogenesis	10	4	0.41	9.67	1.8E-03
Regulation of timing of cell differentiation	13	5	0.54	9.3	5.4E-04
Kinetochore organization	16	6	0.66	9.07	1.7E-04
Regulation of exit from mitosis	14	5	0.58	8.64	7.1E-04
Negative regulation of cell division	14	5	0.58	8.64	7.1E-04
Pulmonary valve morphogenesis	17	6	0.7	8.54	2.2E-04
Regulation of spindle checkpoint	15	5	0.62	8.06	9.2E-04
Attachment of spindle microtubules to kinetochore	18	6	0.74	8.06	2.8E-04
Amino acid import across plasma membrane	15	5	0.62	8.06	9.2E-04
Cytokinetic process	19	6	0.79	7.64	3.6E-04
DNA replication initiation	23	7	0.95	7.36	1.4E-04
Regulation of ubiquitin protein ligase activity	17	5	0.7	7.11	1.5E-03
Notochord development	24	7	0.99	7.05	1.7E-04
Mitotic spindle assembly checkpoint	21	6	0.87	6.91	5.6E-04
G2/M transition of mitotic cell cycle	35	10	1.45	6.91	8.3E-06
Substrate-dependent cell migration	25	7	1.03	6.77	2.1E-04
Positive regulation of cytokinesis	29	8	1.2	6.67	8.3E-05
Positive regulation of cardiac muscle cell proliferation	26	7	1.08	6.51	2.6E-04
Mitotic cytokinesis	64	17	2.65	6.42	1.5E-08
Neuronal stem cell population maintenance	23	6	0.95	6.31	8.4E-04
Epithelial tube branching involved in lung morphogenesis	31	8	1.28	6.24	1.2E-04
Ephrin receptor signaling pathway	31	8	1.28	6.24	1.2E-04
Positive regulation of Notch signaling pathway	40	10	1.65	6.05	2.2E-05
Mitotic metaphase plate congression	40	10	1.65	6.05	2.2E-05
Sprouting angiogenesis	53	13	2.19	5.93	1.6E-06
Positive regulation of focal adhesion assembly	25	6	1.03	5.8	1.2E-03
Olfactory bulb development	38	9	1.57	5.73	8.2E-05
Somatic stem cell division	26	6	1.08	5.58	1.4E-03
Positive regulation of G2/M transition of mitotic cell cycle	26	6	1.08	5.58	1.4E-03
Positive regulation of neural precursor cell proliferation	63	14	2.6	5.37	1.8E-06

Morphogenesis of an epithelial sheet	50	11	2.07	5.32	2.5E-05
Regulation of fibroblast growth factor receptor signaling pathway	32	7	1.32	5.29	7.7E-04
Schwann cell development	33	7	1.36	5.13	9.0E-04
Embryonic digestive tract development	33	7	1.36	5.13	9.0E-04
Embryonic axis specification	38	8	1.57	5.09	4.1E-04
Positive regulation of substrate adhesion-dependent cell spreading	34	7	1.41	4.98	1.1E-03
Eye photoreceptor cell development	34	7	1.41	4.98	1.1E-03
Positive regulation of protein import into nucleus	54	11	2.23	4.93	4.6E-05
Sister chromatid cohesion	40	8	1.65	4.84	5.5E-04
Protein localization to microtubule cytoskeleton	35	7	1.45	4.84	1.2E-03
Cell differentiation involved in kidney development	35	7	1.45	4.84	1.2E-03
Microtubule polymerization or depolymerization	51	10	2.11	4.74	1.3E-04
Glial cell migration	41	8	1.7	4.72	6.4E-04
G1/S transition of mitotic cell cycle	67	13	2.77	4.69	1.5E-05
Cellular response to vascular endothelial growth factor stimulus	37	7	1.53	4.58	1.6E-03
Regulation of neuron migration	48	9	1.98	4.53	3.8E-04
Positive regulation of DNA replication	43	8	1.78	4.5	8.4E-04
Cortical actin cytoskeleton organization	38	7	1.57	4.46	1.8E-03
Ventricular septum morphogenesis	44	8	1.82	4.4	9.6E-04
Aorta development	56	10	2.32	4.32	2.6E-04
Positive regulation of fibroblast proliferation	68	12	2.81	4.27	7.1E-05
Columnar/cuboidal epithelial cell development	57	10	2.36	4.24	2.9E-04
L-amino acid transport	46	8	1.9	4.21	1.2E-03
Negative regulation of osteoblast differentiation	47	8	1.94	4.12	1.4E-03
Oligodendrocyte differentiation	59	10	2.44	4.1	3.8E-04
Myelination	98	16	4.05	3.95	1.1E-05
Anterior/posterior axis specification	50	8	2.07	3.87	2.0E-03
Regulation of cell shape	144	23	5.95	3.86	2.1E-07
Neuroepithelial cell differentiation	63	10	2.6	3.84	6.0E-04
Regulation of cyclin-dependent protein serine/threonine kinase activity	82	13	3.39	3.83	9.6E-05
Cellular response to fibroblast growth factor stimulus	57	9	2.36	3.82	1.1E-03
Negative regulation of cell cycle G2/M phase transition	51	8	2.11	3.79	2.2E-03
Metanephros development	78	12	3.23	3.72	2.3E-04
Meiotic nuclear division	137	21	5.66	3.71	1.2E-06
Osteoblast differentiation	85	13	3.51	3.7	1.3E-04
Centrosome cycle	72	11	2.98	3.69	4.3E-04
Positive regulation of Ras protein signal transduction	59	9	2.44	3.69	1.4E-03
Visual perception	138	21	5.71	3.68	1.4E-06
Nephron tubule development	80	12	3.31	3.63	2.8E-04
Cellular response to calcium ion	74	11	3.06	3.6	5.3E-04
Inner ear morphogenesis	109	16	4.51	3.55	3.6E-05
Negative regulation of canonical Wnt signaling pathway	117	17	4.84	3.51	2.3E-05
Telencephalon cell migration	69	10	2.85	3.51	1.1E-03
Male gonad development	111	16	4.59	3.49	4.4E-05
Inner ear receptor cell differentiation	70	10	2.89	3.45	1.2E-03
Neural precursor cell proliferation	84	12	3.47	3.45	4.2E-04
Chondrocyte differentiation	78	11	3.23	3.41	7.9E-04
Coronary vasculature development	71	10	2.94	3.41	1.4E-03
Cerebral cortex development	107	15	4.42	3.39	1.0E-04
Developmental cell growth	93	13	3.85	3.38	2.9E-04
DNA integrity checkpoint	94	13	3.89	3.34	3.2E-04
Regulation of G1/S transition of mitotic cell cycle	109	15	4.51	3.33	1.2E-04
Plasma membrane organization	73	10	3.02	3.31	1.6E-03
Rho protein signal transduction	75	10	3.1	3.22	2.0E-03
Cell fate specification	75	10	3.1	3.22	2.0E-03
Regulation of stress fiber assembly	84	11	3.47	3.17	1.4E-03
Kidney morphogenesis	84	11	3.47	3.17	1.4E-03
Liver development	100	13	4.13	3.14	5.5E-04

Sensory perception of sound	147	19	6.08	3.13	3.4E-05
Epithelial cell proliferation	93	12	3.85	3.12	9.4E-04
Mesenchymal cell development	86	11	3.56	3.09	1.6E-03
Gland morphogenesis	119	15	4.92	3.05	2.9E-04
Adult locomotory behavior	104	13	4.3	3.02	7.6E-04
Protein-DNA complex assembly	120	15	4.96	3.02	3.1E-04
Cell cycle arrest	88	11	3.64	3.02	1.9E-03
Cellular response to light stimulus	89	11	3.68	2.99	2.1E-03
Establishment of cell polarity	114	14	4.71	2.97	5.7E-04
Positive regulation of protein binding	106	13	4.38	2.97	9.0E-04
Regulation of DNA repair	99	12	4.09	2.93	1.5E-03
Regulation of microtubule cytoskeleton organization	190	23	7.86	2.93	1.4E-05
Neural tube closure	108	13	4.47	2.91	1.1E-03
Developmental growth involved in morphogenesis	125	15	5.17	2.9	4.6E-04
Embryonic limb morphogenesis	142	17	5.87	2.9	2.0E-04
Axon guidance	193	23	7.98	2.88	1.8E-05
Positive regulation of neuron death	111	13	4.59	2.83	1.3E-03
Cellular modified amino acid metabolic process	148	17	6.12	2.78	3.2E-04
Regulation of gliogenesis	131	15	5.42	2.77	7.2E-04
Ameboidal-type cell migration	175	20	7.24	2.76	1.0E-04
Negative regulation of neuron projection development	167	19	6.91	2.75	1.6E-04
Actin filament organization	216	24	8.93	2.69	3.3E-05
Negative regulation of developmental growth	117	13	4.84	2.69	2.0E-03
Cell-substrate adhesion	153	17	6.33	2.69	4.5E-04
Negative regulation of epithelial cell proliferation	138	15	5.71	2.63	1.2E-03
Negative regulation of hemopoiesis	138	15	5.71	2.63	1.2E-03
Regulation of Rho protein signal transduction	129	14	5.33	2.62	1.7E-03
Positive regulation of epithelial cell migration	139	15	5.75	2.61	1.2E-03
Cell-cell adhesion	352	37	14.55	2.54	1.0E-06
Regulation of animal organ morphogenesis	203	21	8.39	2.5	3.6E-04
Potassium ion transport	155	16	6.41	2.5	1.3E-03
Cellular response to oxidative stress	191	19	7.9	2.41	9.4E-04
Regulation of angiogenesis	290	28	11.99	2.34	1.1E-04
Regulation of ERK1 and ERK2 cascade	323	31	13.36	2.32	4.4E-05
Positive regulation of apoptotic process	596	56	24.64	2.27	6.2E-08
Response to wounding	320	30	13.23	2.27	7.9E-05
Regulation of leukocyte differentiation	280	26	11.58	2.25	2.9E-04
Regulation of neuron apoptotic process	248	23	10.25	2.24	7.4E-04
In utero embryonic development	422	38	17.45	2.18	2.7E-05
Negative regulation of cell adhesion	269	24	11.12	2.16	7.7E-04
Positive regulation of neuron projection development	340	30	14.06	2.13	2.2E-04
Positive regulation of protein serine/threonine kinase activity	284	24	11.74	2.04	1.7E-03
Response to peptide	299	25	12.36	2.02	1.5E-03
Muscle tissue development	325	27	13.44	2.01	1.3E-03
Negative regulation of apoptotic process	906	75	37.46	2	5.4E-08
Positive regulation of MAPK cascade	516	42	21.34	1.97	9.5E-05
Negative regulation of transcription by RNA polymerase II	824	67	34.07	1.97	6.3E-07
DNA repair	382	31	15.79	1.96	7.8E-04
Protein phosphorylation	734	59	30.35	1.94	4.1E-06
Muscle structure development	460	35	19.02	1.84	9.9E-04
Positive regulation of transcription by RNA polymerase II	1158	87	47.88	1.82	2.7E-07
Apoptotic process	774	57	32	1.78	5.8E-05
Positive regulation of organelle organization	550	39	22.74	1.71	1.8E-03
Response to drug	729	50	30.14	1.66	9.7E-04

Supplementary Table S6. Full list of overrepresented GO terms of upregulated genes in PN5 Lgr5$^+$ compared to adult Lgr5$^+$. #GO: number of genes in GO term; #Input: number of genes in input list; Exp.: expected number of genes in input list; FE: fold enrichment.

GO term	#GO	#Input	Exp.	FE	P value
Negative regulation of ubiquitin protein ligase activity	10	5	0.38	13.32	1.4E-04
DNA damage response, signal transduction by p53 class mediator resulting in transcription of p21 class mediator	11	5	0.41	12.11	1.9E-04
Ribosomal small subunit assembly	16	6	0.6	9.99	1.0E-04
Regulation of development, heterochronic	15	5	0.56	8.88	6.0E-04
Cytoplasmic translation	50	16	1.88	8.52	1.2E-09
Ribosomal large subunit assembly	34	9	1.28	7.05	1.9E-05
Sympathetic nervous system development	23	6	0.86	6.95	5.1E-04
ATP-dependent chromatin remodeling	36	8	1.35	5.92	1.6E-04
Positive regulation of Notch signaling pathway	40	8	1.5	5.33	3.0E-04
Ventricular septum morphogenesis	44	8	1.65	4.84	5.2E-04
Mitotic cell cycle phase transition	118	20	4.43	4.51	1.3E-07
Lens development in camera-type eye	65	11	2.44	4.51	8.7E-05
Cell cycle G1/S phase transition	68	10	2.55	3.92	5.0E-04
Retina development in camera-type eye	137	18	5.14	3.5	1.3E-05
Neural tube closure	108	14	4.06	3.45	1.4E-04
Digestive tract development	114	14	4.28	3.27	2.3E-04
Telencephalon development	234	27	8.79	3.07	1.0E-06
Camera-type eye morphogenesis	122	14	4.58	3.06	4.2E-04
Axon guidance	193	21	7.25	2.9	3.5E-05
Kidney development	246	26	9.24	2.81	7.0E-06
DNA conformation change	166	17	6.23	2.73	3.7E-04
Negative regulation of neuron projection development	167	17	6.27	2.71	3.9E-04
rRNA processing	170	17	6.38	2.66	4.7E-04
Central nervous system neuron differentiation	192	19	7.21	2.64	2.5E-04
Negative regulation of binding	162	16	6.08	2.63	7.7E-04
Regulation of axonogenesis	199	19	7.47	2.54	3.8E-04
Positive regulation of cell cycle process	231	22	8.67	2.54	2.3E-04
Lymphocyte differentiation	253	24	9.5	2.53	1.1E-04
Regulation of Ras protein signal transduction	211	20	7.92	2.52	3.0E-04
Cell fate commitment	249	22	9.35	2.35	4.2E-04
Cell division	464	38	17.42	2.18	2.7E-05
Epithelial cell differentiation	499	40	18.74	2.13	1.9E-05
Blood vessel morphogenesis	407	32	15.28	2.09	2.3E-04
Cell proliferation	560	44	21.03	2.09	1.2E-05
Actin cytoskeleton organization	442	33	16.6	1.99	4.1E-04
Embryonic organ development	466	34	17.5	1.94	5.7E-04
Positive regulation of cell proliferation	949	68	35.63	1.91	1.1E-06
Regulation of cell adhesion	647	43	24.29	1.77	5.6E-04
Negative regulation of cell proliferation	651	43	24.44	1.76	5.9E-04
Negative regulation of transcription by RNA polymerase II	824	54	30.94	1.75	1.4E-04
Negative regulation of signal transduction	1140	73	42.81	1.71	2.3E-05
Positive regulation of cell differentiation	1042	66	39.13	1.69	6.6E-05
Regulation of locomotion	954	58	35.82	1.62	5.3E-04
Positive regulation of transcription, DNA-templated	1465	87	55.01	1.58	5.1E-05
Transcription, DNA-templated	1890	104	70.97	1.47	1.5E-04

Supplementary Table S7. Full list of overrepresented GO terms of downregulated genes in PN5 Lgr5$^+$ compared to adult Lgr5$^+$. #GO: number of genes in GO term; #Input: number of genes in input list; Exp.: expected number of genes in input list; FE: fold enrichment.

GO term	#GO	#Input	Exp.	FE	P value
Calcium ion export	10	5	0.52	9.61	5.8E-04
Membrane repolarization	15	7	0.78	8.96	6.3E-05
Cerebellar Purkinje cell differentiation	13	6	0.68	8.87	2.3E-04
Sodium ion export across plasma membrane	11	5	0.57	8.73	8.1E-04
Relaxation of muscle	18	7	0.94	7.47	1.6E-04
G-protein coupled glutamate receptor signaling pathway	16	6	0.83	7.2	5.5E-04
CGMP metabolic process	17	6	0.88	6.78	7.1E-04
Long term synaptic depression	18	6	0.94	6.4	9.0E-04
Neuron cellular homeostasis	18	6	0.94	6.4	9.0E-04
Phototransduction	28	9	1.46	6.17	6.1E-05
Detection of visible light	29	9	1.51	5.96	7.6E-05
Synaptic transmission, glutamatergic	36	10	1.87	5.34	6.7E-05
Synaptic vesicle endocytosis	29	8	1.51	5.3	3.7E-04
Cholesterol biosynthetic process	30	8	1.56	5.12	4.5E-04
Cardiac muscle cell contraction	30	8	1.56	5.12	4.5E-04
Potassium ion import	38	10	1.98	5.06	9.9E-05
Positive regulation of potassium ion transmembrane transport	36	9	1.87	4.8	3.0E-04
Chemical synaptic transmission, postsynaptic	45	11	2.34	4.7	7.9E-05
Calcium ion import	41	10	2.13	4.69	1.7E-04
Regulation of long-term synaptic potentiation	43	10	2.24	4.47	2.4E-04
Regulation of long-term neuronal synaptic plasticity	35	8	1.82	4.39	1.1E-03
Action potential	86	19	4.48	4.24	8.6E-07
Response to pH	41	9	2.13	4.22	6.9E-04
Positive regulation of synapse assembly	73	16	3.8	4.21	6.7E-06
Synaptic vesicle exocytosis	56	12	2.92	4.12	1.1E-04
Visual learning	66	14	3.44	4.07	3.4E-05
Synapse assembly	58	12	3.02	3.97	1.5E-04
Inorganic cation import across plasma membrane	44	9	2.29	3.93	1.1E-03
Protein localization to synapse	44	9	2.29	3.93	1.1E-03
Adult walking behavior	44	9	2.29	3.93	1.1E-03
Regulation of postsynaptic membrane potential	59	12	3.07	3.91	1.7E-04
Circadian regulation of gene expression	60	12	3.12	3.84	2.0E-04
Regulation of potassium ion transmembrane transporter activity	55	11	2.86	3.84	3.6E-04
Transmission of nerve impulse	65	13	3.38	3.84	1.1E-04
Regulation of cAMP-mediated signaling	62	12	3.23	3.72	2.6E-04
Calcium ion transmembrane transport	135	26	7.03	3.7	9.7E-08
Cellular response to calcium ion	74	14	3.85	3.63	1.0E-04
Positive regulation of regulated secretory pathway	53	10	2.76	3.62	9.9E-04
Memory	139	26	7.24	3.59	1.6E-07
Regulation of catecholamine secretion	71	13	3.7	3.52	2.4E-04
Sensory perception of pain	100	18	5.21	3.46	2.0E-05
Neuromuscular process	127	22	6.61	3.33	4.3E-06
Regulation of synaptic transmission, glutamatergic	73	12	3.8	3.16	9.5E-04
Visual perception	138	22	7.18	3.06	1.4E-05
Positive regulation of synaptic transmission	172	26	8.95	2.9	5.5E-06
Amino acid transport	100	15	5.21	2.88	5.5E-04
Dendrite development	121	18	6.3	2.86	1.8E-04
Response to purine-containing compound	108	16	5.62	2.85	4.1E-04
Response to mechanical stimulus	143	21	7.44	2.82	6.2E-05
Regulation of heart contraction	167	24	8.69	2.76	2.6E-05
Regulation of circadian rhythm	105	15	5.47	2.74	8.6E-04
Detection of stimulus involved in sensory perception	127	17	6.61	2.57	7.8E-04
Lipid homeostasis	128	17	6.66	2.55	8.4E-04
Regulation of calcium ion transmembrane transport	144	19	7.5	2.53	7.1E-04

Regulation of cation channel activity	153	20	7.96	2.51	5.2E-04
Regulation of endocytosis	242	30	12.6	2.38	5.0E-05
Regulation of dendrite development	178	22	9.27	2.37	4.3E-04
Adenylate cyclase-modulating G-protein coupled receptor signaling pathway	188	23	9.79	2.35	3.5E-04
Regulation of axonogenesis	199	24	10.36	2.32	4.5E-04
Negative regulation of neuron apoptotic process	166	20	8.64	2.31	1.0E-03
Positive regulation of neuron projection development	340	39	17.7	2.2	1.9E-05
Positive regulation of ERK1 and ERK2 cascade	245	28	12.75	2.2	3.3E-04
Regulation of cytosolic calcium ion concentration	322	35	16.76	2.09	1.1E-04
Second-messenger-mediated signaling	282	30	14.68	2.04	5.2E-04
Cellular response to organonitrogen compound	379	40	19.73	2.03	8.4E-05
Negative regulation of neurogenesis	314	33	16.35	2.02	3.8E-04
Regulation of hormone secretion	288	30	14.99	2	9.3E-04
Axonogenesis	313	32	16.29	1.96	6.3E-04
Protein complex oligomerization	537	53	27.95	1.9	2.8E-05
Cellular response to organic cyclic compound	363	35	18.9	1.85	9.8E-04
Response to drug	729	64	37.95	1.69	1.1E-04
Regulation of cellular localization	825	72	42.95	1.68	5.3E-05
Response to hormone	589	51	30.66	1.66	8.0E-04
Cellular response to oxygen-containing compound	784	65	40.81	1.59	4.8E-04
Cellular lipid metabolic process	774	64	40.29	1.59	6.0E-04
Cell adhesion	732	60	38.1	1.57	1.1E-03
Negative regulation of signal transduction	1140	91	59.34	1.53	1.4E-04
